# Effects of pH on the Pathogenicity of *Escherichia coli* and *Klebsiella pneumoniae* on the Kidney: In Vitro and In Vivo Studies

**DOI:** 10.3390/ijms25147925

**Published:** 2024-07-19

**Authors:** Soraya Herrera-Espejo, José Luis Domínguez-Miranda, Juan Ignacio Rodríguez-Mogollo, Jerónimo Pachón, Elisa Cordero, María Eugenia Pachón-Ibáñez

**Affiliations:** 1 Clinical Unit of Infectious Diseases, Microbiology and Parasitology, Institute of Biomedicine of Seville (IBiS), Virgen del Rocio University Hospital/CSIC/University of Seville, 41013 Seville, Spain; sherrera-ibis@us.es (S.H.-E.); juanignaciorrmm14@gmail.com (J.I.R.-M.); mpachon-ibis@us.es (M.E.P.-I.); 2 Department of Pathology, Virgen de Rocío University Hospital, 41013 Seville, Spain; jluismiranda77@gmail.com; 3 Institute of Biomedicine of Seville (IBiS), Virgen del Rocio University Hospital/CSIC/University of Seville, 41013 Seville, Spain; 4 Department of Medicine, School of Medicine, University of Seville, 41004 Seville, Spain; 5 CIBER de Enfermedades Infecciosas (CIBERINFEC), Instituto de Salud Carlos III, 28029 Madrid, Spain

**Keywords:** urinary tract infections, urine pH, *Escherichia coli*, *Klebsiella pneumoniae*, experimental murine model

## Abstract

Urine pH reflects the functional integrity of the body and may influence the virulence of uropathogenic *Escherichia coli* and *Klebsiella pneumoniae*, the main causes of urinary tract infections (UTIs). This study evaluated the effects of acidic pH on the pathogenicity of uropathogenic *E. coli* and *K. pneumoniae* strains, in vitro and in vivo. Four uropathogenic *E. coli* and four *K. pneumoniae* strains were used. Biofilm formation, growth competition indices, motility, and adhesion and invasion of human renal cells were analyzed in media with acidic, neutral, and alkaline pH. A murine lower UTI model was used, with urine adjusted to acidic, neutral, or alkaline pH. At acidic pH, *E. coli* and *K. pneumoniae* exhibited higher bacterial concentrations in the kidneys and systemic symptoms, including bacteremia. Alkaline urine pH did not affect bacterial concentrations of any strain. In mice with UTIs caused by *E. coli* Nu14 and *K. pneumoniae* HUVR42 and acidic urine pH, histopathological studies of the kidneys showed acute inflammation affecting the urothelium and renal parenchyma, which are traits of acute pyelonephritis. These results indicate that acidic pH could increase the pathogenicity of *E. coli* and *K. pneumoniae* in murine models of lower UTI, promoting renal infection and acute inflammation.

## 1. Introduction

Urine reflects the functional integrity of the body and often acts as an early indicator of both pathological and physiological changes [1,2]. Due to the absence of homeostatic mechanisms in urine, pH responds dynamically to bodily changes, making it a crucial biomarker [3]. With a normal range of pH 4.5 to 8.0 [4], urine pH can significantly affect the virulence factors and metabolic activities of uropathogenic microorganisms [1], assisting them in colonizing the urinary system and evading the immune system [5].

*Escherichia coli* and *Klebsiella pneumoniae* are widely recognized pathogens responsible for urinary tract infections (UTIs) and are the first and second most common species found in its etiology [6,7]. Uropathogenic strains typically ascend from the colonized urethra or perineal area to the bladder, facilitated by flagella and fimbriae, where they adhere to and invade the superficial bladder cells.

Several virulence factors have been identified in uropathogenic *E. coli* (UPEC), including flagella [8] and adhesin-like Type I fimbriae [9] alongside Dr adhesion [10], S fimbriae [11], and F1C fimbriae [12]. In addition, uropathogenic *K. pneumoniae* employ two types of fimbrial adhesins, Type 1 and Type 3, for colonization [13]. Type 1 fimbriae bind to mannose receptors, facilitating bladder cell invasion [14], and Type 3 fimbriae play an important role in biofilm aggregation on medical devices, such as catheters [14].

In a cohort study of kidney transplant recipients with an episode of UTI, the acidic urine pH at diagnosis was not associated with the microbiological efficacy of the antimicrobial therapy but was associated with symptomatic UTI episodes at one-month follow-up, specifically when caused by *E. coli* [15]. The pH of the local microenvironment plays a critical role in determining the potential virulence of uropathogenic bacteria [16]. Acidic pH conditions have been implicated in the modulation of bacterial adhesion to host cells, tissue invasion, and biofilm formation [17]. Although the specific mechanisms underlying these effects remain elusive, it is postulated that acidic pH may alter bacterial gene expression, leading to the differential regulation of production of virulence factors. Further investigations are warranted to elucidate the interplay between pH dynamics and bacterial pathogenicity in UTI. Therefore, this study aimed to assess, in vitro and in vivo, the impact of acidic pH on the pathogenicity of uropathogenic strains.

## 2. Results

### 2.1. Characterization of Test Strains

#### 2.1.1. Multi-Locus Sequence Type

Uropathogenic *E. coli* Nu14 and two isogenic strains, *E. coli* Nu14 carrying a point mutation in *gyrA* (D87G) and a *glpT* missense mutation, belonged to the same clone ST95. *E. coli* HUVR94 belonged to clone ST9744. The uropathogenic *K. pneumoniae* strains were clonally unrelated: *K. pneumoniae* HUVR42 belonged to ST2701, *K. pneumoniae* HUVR5 to ST466, *K. pneumoniae* HUVR110 to ST342, and *K. pneumoniae* to HUVR91 ST37 (Table 1).

#### 2.1.2. In Vitro Growth Curves and Competition Indices

Independent of the media tested, on a Luria-Bertani (LB) plate or in filter-sterilized normal human urine, in competition assays, *E. coli* HUVR94 showed higher growth than the other strains, irrespective of pH. Among the three isogenic strains, *E. coli* Nu14 with the *glpT* missense mutation showed a higher rate of competition at neutral pH than the other two strains, with minimal changes at both acidic and alkaline pH. Between *E. coli* Nu14 and *E. coli gyrA* D87G, *E. coli* Nu14 showed higher bacterial fitness at neutral and acidic pH than at alkaline pH (Supplementary Figures S1 and S2).

For *K. pneumoniae*, *K. pneumoniae* strain HUVR42 showed higher bacterial fitness than the resistant strains regardless of the pH and media tested. In competition indices performed in MHB or in filter-sterilized human urine, no differences were found when comparing the pH values except between HUVR5 and HUVR110, where HUVR5 showed an advantage at neutral or alkaline pH and HUVR110 at acidic pH (Supplementary Figures S3 and S4).

### 2.2. In Vitro Studies

#### 2.2.1. Biofilm Assay

**Figure 1:**
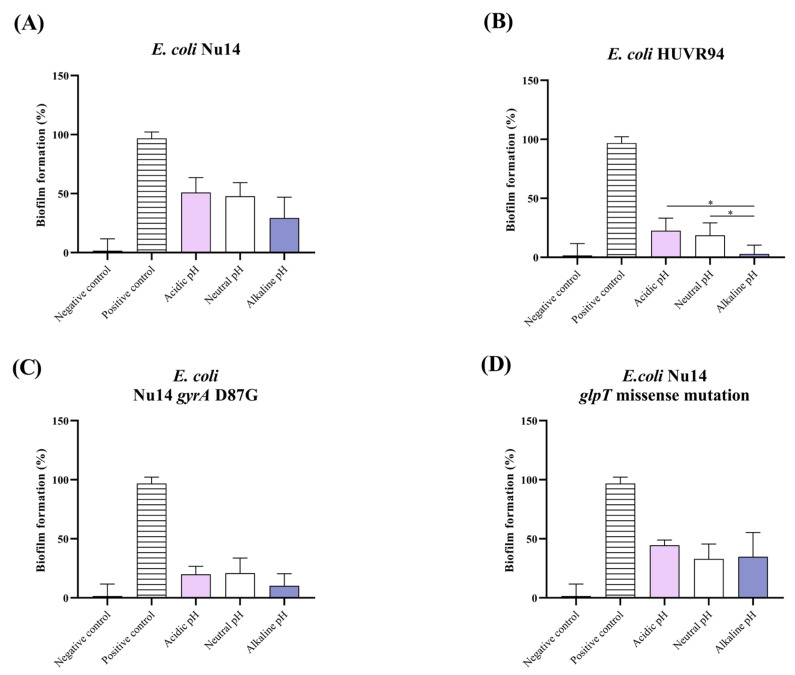
Biofilm formation at different media pH of *Escherichia coli* Nu14, HUVR94 clinical isolate, Nu14 *gyrA* D87G, and Nu14 *glpT* missense mutation strains. (**A**) *E. coli* Nu14; (**B**) *E. coli* HUVR94 clinical isolate; (**C**) *E. coli* Nu14 *gyrA* D87G; (**D**) *E. coli* Nu14 *glpT* missense mutation. Negative control: LB (Luria-Bertani broth); positive control: *E. coli* ATCC25922; pink bar: acidic LB broth; white bar: neutral LB broth; purple bar: alkaline LB broth. *: *p* < 0.05.

**Figure 2:**
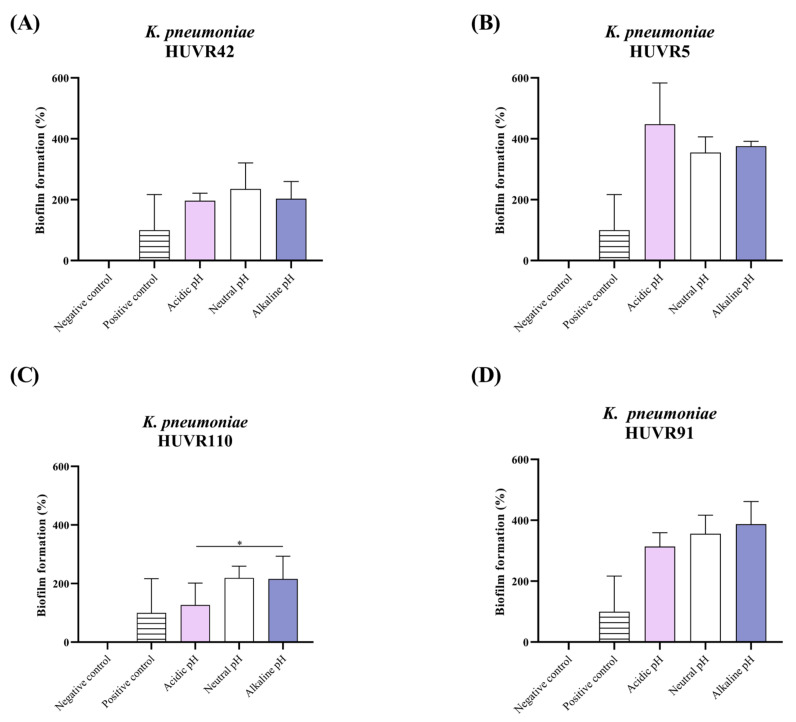
Biofilm formation at different media pH of *Klebsiella pneumoniae* HUVR42, HUVR5, HUVR91, and HUVR110 strains. (**A**) *K. pneumoniae* HUVR42; (**B**) *K. pneumoniae* HUVR5; (**C**) *K. pneumoniae* HUVR91; (**D**) *K. pneumoniae* HUVR110. Negative control: LB (Luria-Bertani broth); positive control: *K. pneumoniae* CECT997; pink bar: acidic LB broth; white bar: neutral LB broth; purple bar: alkaline LB broth. *: *p* < 0.05.

For the four UPEC strains, biofilm formation rates at neutral pH ranged from 20% to 50% compared to the positive control (Figure 1). In contrast, the uropathogenic *K. pneumoniae* strains exhibited higher biofilm formation, ranging from 200% to 380%, compared to the positive control (Figure 2).

At acidic pH, similar biofilm formation rates were observed at neutral pH for the four UPEC and *K. pneumoniae* strains. Notably, *K. pneumoniae* HUVR5 showed a higher biofilm formation rate at an acidic pH. At alkaline pH, three (Nu14, Nu14 *gyrA* D78G, and HUVR94) *E. coli* strains exhibited lower biofilm formation rates than at neutral pH, which was significant for *E. coli* HUVR94. In contrast, for *K. pneumoniae* strains, no differences were observed between alkaline and neutral pH conditions.

#### 2.2.2. Motility Assay

All UPEC strains showed a decreased motility (below 40%) compared to positive controls in both an LB plate or in filter-sterilized normal human urine containing 0.3% agarose. Independent of the media tested, motility was unaffected by acidic or alkaline media compared with neutral pH media (Supplementary Figure S5A,B). For *K. pneumoniae* strains, motility at neutral pH ranged between 50% and 75% compared to the positive control in both media; although motility showed strain-dependent results, no significant differences were observed. When comparing acidic and neutral pH, HUVR110 showed higher motility, HUVR42 and HUVR91 showed similar motility, and HUVR5 showed the lowest motility. However, HUVR5 showed the higher motility in the acidic filter-sterilized human urine motility plate than at neutral. At alkaline and neutral pH, HUVR42 and HUVR 110 exhibited higher motility, HUVR91 exhibited similar motility, and HUVR5 exhibited lower motility in both media tested.

#### 2.2.3. Adherence to and Invasion of Embryonic Kidney Epithelial Cells (Line HEK-293)

**Figure 3:**
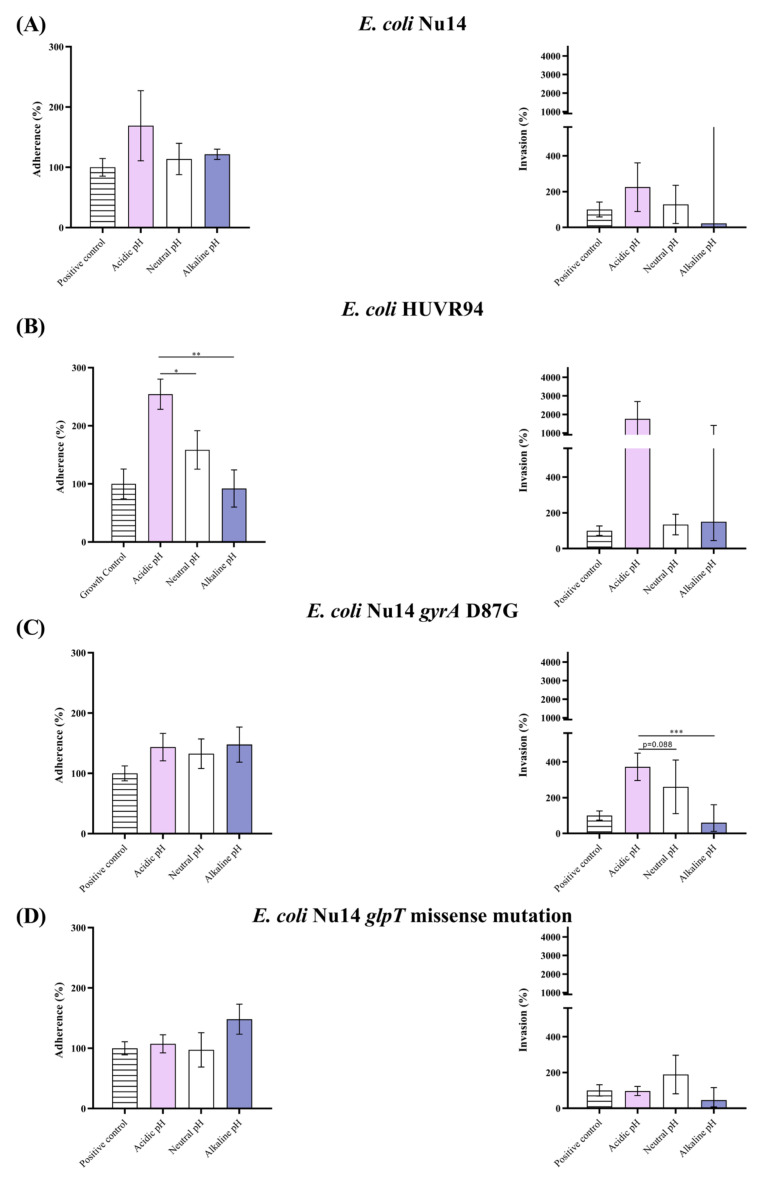
Adherence to and invasion of embryonic kidney epithelial cells (line HEK-293) by *E. coli* Nu14, HUVR94 clinical isolate, Nu14 *gyrA* D87G, and Nu14 *glpT* missense mutation strains at different media pH. Adherence (left) and invasion (right) of (**A**) *E. coli* Nu14; (**B**) *E. coli* HUVR94 clinical isolate; (**C**) *E. coli* Nu14 *gyrA* D87G; (**D**) *E. coli* Nu14 *glpT* missense mutation into human embryonic kidney cells. Stripped bar: growth control (bacterial growth in DMEM at standard pH 7.4); pink bar: acidic pH; white bar: neutral pH; purple bar: alkaline pH. *: *p* < 0.05; **: *p* < 0.01; ***: *p* < 0.001.

At neutral pH, adherence rates to HEK-293 cells were approximately 100% for all UPEC strains, and invasion rates ranged from 150 to 300% compared to growth control Dulbecco modified Eagle medium (DMEM) (Figure 3). At acidic pH, adherence and invasion rates were higher in two strains (*E. coli* Nu14 and *E. coli* Nu14 *gyrA* D87G) and three strains (*E. coli* Nu14, *E. coli* HUVR94, and *E. coli* Nu14 *gyrA* D87G), respectively, than at neutral pH. When comparing alkaline to neutral pH, the three strains exhibited higher adherence but lower invasion (*E. coli* Nu14, *E. coli* Nu14 *gyrA* D87G, and *E. coli* Nu14 *glpT*).

**Figure 4:**
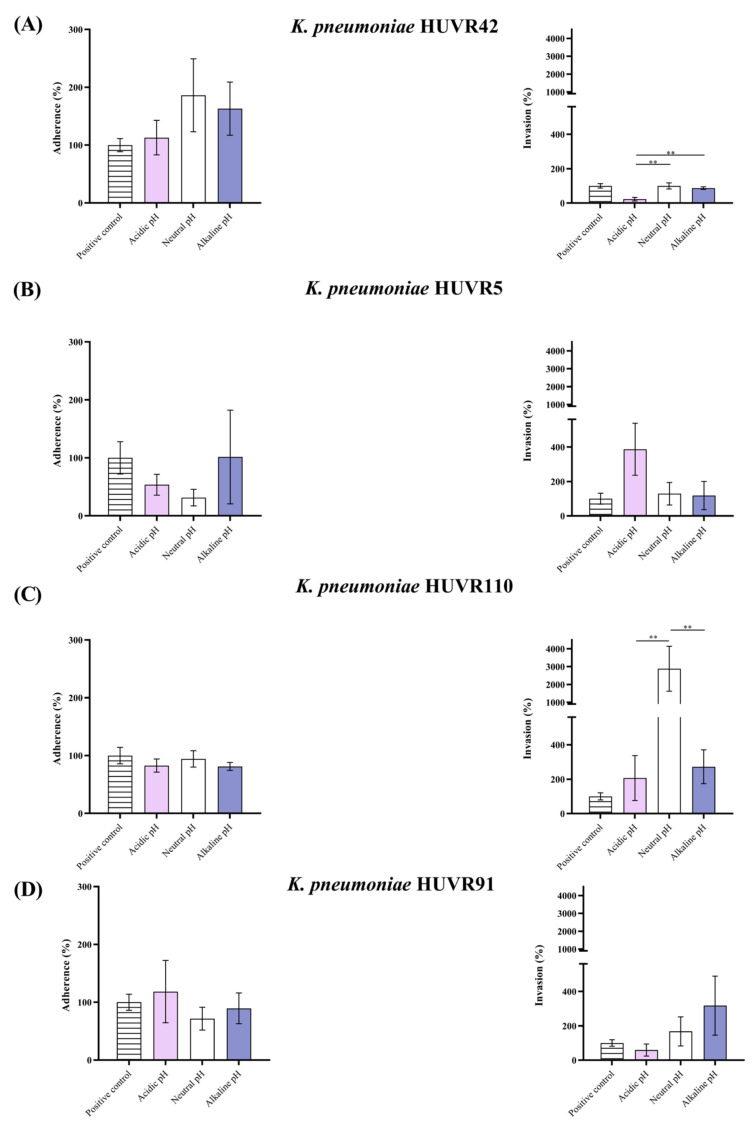
Adherence to and invasion of embryonic kidney epithelial cells (line HEK-293) by *Klebsiella pneumoniae* HUVR42, HUVR5, HUVR91, and HUVR110 strains at different media pH. Adherence (left) and invasion (right) of (**A**) *K. pneumoniae* HUVR42; (**B**) *K. pneumoniae* HUVR5; (**C**) *K. pneumoniae* HUVR91; (**D**) *K. pneumoniae* HUVR110 into human embryonic kidney cells. Stripped bar: growth control (bacterial growth in DMEM at standard pH 7.4); pink bar: acidic pH; white bar: neutral pH; purple bar: alkaline pH. **: *p* < 0.01.

Regarding *K. pneumoniae* strains, adherence and invasion rates at neutral pH were more heterogeneous among the studied strains (Figure 4). At acidic or alkaline pH, two strains (HUVR5 and HUVR91) showed higher adherence to HEK-293 cells than at neutral pH. However, the cell invasion of HEK-293 was lower at acidic pH in the strains HUVR49, HUVR110, and HUVR91 than at neutral pH.

### 2.3. In Vivo Studies

#### 2.3.1. Bacterial Concentrations in Tissues and Blood, Systemic Symptoms, and Mortality in UTI Murine Models by *E. coli* and *K. pneumoniae* at Different Urine pHs

No differences were observed in bacterial kidney and bladder concentration between neutral and alkaline urine pH (Supplementary Table S1). To follow the 3Rs rules [20], the in vivo model of lower UTI was performed at neutral and acidic urine pH with all strains and at alkaline urine pH only with *E. coli* Nu14 and *K. pneumoniae* HUVR42.

In the UTI model with immunocompetent mice, at acidic urine pH, infected mice showed higher *E. coli* bacterial concentrations in kidneys than infected mice with neutral urine pH. Moreover, some mice with an acidic urine pH died (20%) when infected with Nu14 and Nu14 *glpT* missense mutation strains and presented systemic signs, with weight loss (up to 8.4%), bloodstream infection (BSI; range 20–80%), and fever. Regarding bacterial concentration in the bladder, *E. coli* Nu14-infected mice showed lower bacterial concentrations with acidic urine pH than those with neutral urine pH (Table 2). In mice with alkaline urine pH infected with *E. coli* Nu14, there were no differences in bacterial concentrations in tissues and urine compared to mice with neutral urine pH and without BSI or mortality (Supplementary Table S1).

Similarly, in the case of the UTI model caused by *K. pneumoniae*, mice with acidic urine pH had higher kidney bacterial concentrations than mice with neutral urine pH for all studied strains. Systemic signs without mortality were observed at acidic urine pH, with weight loss (up to 7.9%) and BSI (67%) in two (HUVR42 and HUVR91) out of four strains. Acidic urine pH infected mice showed lower bacterial concentrations in the bladder and urine than in mice with neutral urine pH for four and three strains, respectively (Table 3). Experiments in mice with alkaline urine pH were performed for *K. pneumoniae* HUVR42, with no differences in bacterial concentrations compared to mice with neutral urine pH and with no BSI or mortality (Supplementary Table S1).

In the *E. coli* UTI model in immunocompromised mice, animals with acidic urine pH had higher bacterial concentrations in kidneys than mice with neutral urine pH for three (Nu14, HUVR94, and Nu14 *glpT* missense mutation) of the strains, reaching similar bacterial concentrations in kidneys to those in immunocompetent mice except for HUVR94 (7.80 ± 0.48 and 4.91 ± 0.51 Log_10_ CFU/g, respectively, *p* < 0.05). Although mortality was not observed, systemic signs (fever and BSI ranging from 33% to 100%) were detected in all four strains (Table 2 and Supplementary Figure S6).

In the *K. pneumoniae* UTI model in immunocompromised animals, mice with acidic urine had higher kidney bacterial concentrations than those with neutral urine pH for all three strains. No mortality was observed in this model either, but there were systemic signs (fever, weight loss, and BSI in three strains, ranging from 25% to 33%). No differences in bacterial concentrations were observed in the bladder or urine (Table 3 and Supplementary Figure S6).

#### 2.3.2. Kidneys’ Histopathology Findings in UTI Murine Models at Different Urine pH

In mice with acidic urine and *E. coli* Nu14 UTI, histopathological studies of the kidneys (Supplementary Figure S7) showed acute tubular inflammatory cellularity caused by neutrophils, affecting the urothelium and renal parenchyma, confirming acute pyelonephritis. The kidneys of the mice with neutral or alkaline urine pH did not exhibit any inflammatory cellularity. Likewise, histopathological studies of kidneys from mice with acidic or alkaline urine and *K. pneumoniae* HUVR42 UTI (Supplementary Figure S8) showed acute inflammatory cellularity by neutrophils and infiltrates affecting the urothelium and renal parenchyma, confirming acute pyelonephritis, whereas kidneys from infected mice with neutral urine pH did not show inflammatory cellularity.

## 3. Discussion

The present study showed that acidic pH increases the pathogenicity of uropathogenic *E. coli* and *K. pneumoniae* strains. In both in vivo ITU models, mice with acidic urine pH had higher bacterial concentrations in the kidneys together with the acute inflammatory cellularity infiltrates affecting the urothelium and renal parenchyma, shown by the histopathological studies. Similar results were observed in the immunocompromised mice. To the best of our knowledge, this is the first in vivo evidence of the effect of acidic urine pH on the virulence of uropathogenic pathogens. In vitro, adherence to and invasion of embryonic kidney epithelial cells by *E. coli* increased at acidic pH compared to neutral pH in two (*E. coli* Nu14 and HUVR94) and three (*E. coli* Nu14, HUVR94 and *gyrA* D87G) out of the four strains assessed, respectively. For the *K. pneumoniae* strains, two (HUVR5 and HUVR91) and one (HUVR5) of the four strains also showed higher adherence to and invasion of cells at acidic pH. There were no consistent differences in biofilm formation, bacterial fitness, or motility of *E. coli* and *K. pneumoniae* strains with respect to pH.

The in vivo experiments showed that infected immunocompetent and immunosuppressed mice with acidic urine pH had reduced bacterial concentrations in the bladder with one *E. coli* and the four *K. pneumoniae* strains compared to those in mice with neutral urine pH. This was probably because of a sub-optimal environment affecting bacterial growth [21,22,23]. An in vitro study reported that glucose in urine may stimulate uropathogenic bacteria, increasing the production of biomass, biofilm formation, and metabolic activity [1], which is why for the neutral urine pH mice model of the present study, only glucose was supplemented to successfully infect the lower urinary tract.

Peng et al. [24] showed in a culture of an M-1 immortalized mouse collecting duct cell line that acid loading induced antimicrobial peptide mRNA expression and M-1 cell resistance to uropathogenic *E. coli* infection, which differs from our findings regarding the adherence to and invasion of urinary cells. However, Peng et al. [24] used a mildly acidic pH of 6.8, whereas the acidic conditions used in this study had a pH between 5 and 5.5. Moreover, Peng et al. used a differentiated mouse renal cell line, whereas the present study used a human pluripotent kidney cell line (HEK cells).

Although an acidic urine pH seems to reduce lower UTIs, as shown by the bladder and urine bacterial concentrations in our experiments, the data indicate that both *E. coli* and *K. pneumoniae* are capable of ascending to the kidneys and causing the development of pyelonephritis, as confirmed by microbiological and histopathological analyses, accompanied by systemic signs such as fever, weight loss, bacteremia, and even mortality. This observation suggests that metabolic acidosis may predispose individuals to acute pyelonephritis [15]. Defects in carbonic anhydrase have been associated with renal tubular acidosis; thus, carbonic anhydrase 2-deficient mice, characterized by metabolic acidosis, exhibit high bacterial concentrations in the kidneys after transurethral inoculation with uropathogenic *E. coli* [25,26].

Our findings strongly suggest that acidic urine increases the pathogenicity of uropathogenic strains and causes pyelonephritis. These results support the hypothesis that an acidic pH modulates bacterial gene expression. Notably, many virulence factors are pH-sensitive, such as siderocalin, which plays a major role in the defense against host immune responses [27], fimbriae [28], and efflux pumps associated with biofilm formation and quorum sensing regulation [29]. Additionally, a two-component system (PhoQ/PhoP) has been previously described in *E. coli*. PhoP, overexpressed under acidic conditions, regulates several virulence factors such as flagella or biofilm production [30]. Consequently, various virulence assays were conducted. Among the uropathogenic *E. coli* strains studied, three of four belonged to the prevalent sequence type ST95 causing UTIs [31,32]. Conversely, uropathogenic *K. pneumoniae* strains did not correspond to the most common sequence types linked to UTIs. However, two out of four *K. pneumoniae* strains belonged to sequence types previously identified as virulent, exhibited carbapenem resistance, and were implicated in neonatal sepsis [33,34]. In addition, these strains were reported to persist in natural reservoirs [35]. 

The in vitro studies of growth kinetics, competition indices, motility, and biofilm formation showed no relevant changes at different pH conditions for *E. coli* or for *K. pneumoniae*. The roles of bacterial adhesion to host cells and biofilm formation in the pathogenicity of UTIs have been widely documented [36,37]. Previous studies have reported also that *Streptococcus mutans* strains display an enhanced capacity for biofilm formation in acidic environments, suggesting their potential adaptation to survive external aggressions [38]. However, the strains tested in the present study did not show changes in the biofilm formation, consistent with the results found in the in vivo studies.

Adherence and invasion assays demonstrated a strain-dependent response to acidic pH, with most *E. coli* strains exhibiting enhanced cell invasion under acidic conditions, whereas *K. pneumoniae* invasion results were heterogeneous. Valdebenito et al. [39] reported that UPEC have specific iron acquisition mechanisms with greater affinity and expression at acidic pH, implying a possible link between urinary pH and bacterial virulence. This observation could also explain the higher bacterial concentration in the kidneys under acidic urine pH, where iron levels are typically higher than those in urine [40]. Despite these results, the precise mechanisms underlying the observed differences in pathogenicity under acidic urine pH conditions remain unclear, and further studies are required to fully understand the complex interplay between pH dynamics and bacterial virulence. Transcriptomic analysis can provide valuable insights into the metabolic pathways affected by pH variation, shedding light on the underlying molecular mechanisms.

Our study had several limitations. Murine models used may not fully reflect the complexity of UTIs in humans. Although various aspects of bacterial virulence have been explored, many other unexplored factors contribute to the development of infection. Hence, it is important to further investigate the molecular mechanisms underlying the effect of urinary pH on bacterial virulence and explore how other factors in the urinary microenvironment might influence this process. However, the strengths of this study lie in the utilization of in vivo experimental models in immunocompetent and immunosuppressed mice with the two most common pathogens causing UTI, using well-characterized strains to confirm that they showed no differences in their pathogenicity and virulence other than that which may be caused by the acidic pH.

In conclusion, the results of the present study highlight that acidic urinary pH affects the pathogenicity of uropathogenic *E. coli* and *K. pneumoniae* favoring the development of pyelonephritis. These findings may have important implications for clinical practice, recognizing the role of urinary pH in bacterial virulence and supporting the evaluation of new management strategies, such as the modulation of urine pH as a complementary approach for treating patients with recurrent UTIs.

## 4. Materials and Methods

### 4.1. Bacterial Strains

Four uropathogenic *E. coli* and *K. pneumoniae* strains were used: *E. coli* Nu14 [41], *E. coli* HUVR94, *E. coli* Nu14 *gyrA* D87G [42], and *E. coli* Nu14 with a *glpT* missense mutation [43]; *K. pneumoniae* HUVR42, *K. pneumoniae* HUVR5, *K. pneumoniae* HUVR110, and *K. pneumoniae* HUVR91 [15]. Antibiotic susceptibility patterns are detailed in Table 1.

### 4.2. In Vitro Studies

In vitro experiments were performed in triplicate on different days.

#### 4.2.1. Antimicrobial Susceptibility Assay and Multi-Locus Sequence Typing

Ciprofloxacin, fosfomycin, and amikacin were purchased as standard powders (Sigma-Aldrich, Madrid, Spain). Minimum inhibitory concentrations (MICs) of ciprofloxacin and amikacin was determined by the microdilution method. Increasing concentrations of ciprofloxacin (from 0.01 to 8 mg/L) were tested with a starting inoculum of 5 × 10^5^ CFU/mL. Fosfomycin susceptibility assays were carried out by the agar diffusion method. Aliquots of MHB-agar (Sigma-Aldrich, Madrid, Spain) were supplemented with glucose-6-phosphate (25 mg/L) and increasing concentrations of fosfomycin (from 0.12 to 1024 mg/L). Aliquots were plated and gelled, then 2 µL of inoculum (final concentration of 5 × 10^5^ CFU/mL) was plated and incubated overnight at 37 °C.

Polymerase chain reaction (PCR) was used for the multi-locus typing. Primers targeting seven housekeeping genes for *E. coli* [18] and seven housekeeping genes for *K. pneumoniae* [19] were purchased from Thermo Fischer Scientific (Waltham, MA, USA; sequences detailed in Supplementary Table S2). The PCR amplification conditions have been described previously [18]. PCR amplicons were purified using ExoSAP-IT reagent (Applied Biosystems, Foster City, CA, USA) and subsequently sequenced by Sanger using an ABI3500 Genetic Analyzer (Applied Biosystems), and data were compiled through the website hosted at the Center of Genomic Epidemiology (CGE) [44].

#### 4.2.2. In Vitro Growth Curves and Competition Indices

Growth curves and competition indices (CI) were generated at acidic, neutral, and alkaline pH. Each strain (5 × 10^5^ CFU/mL) was grown at 37 °C in 10 mL of MHB (Sigma-Aldrich) in an adjusted plate or in filter-sterilized normal human urine immediately prior to the start of the assays to obtain acidic pH (pH = 5) or alkaline pH (pH = 8) by adding 0.012% (*v*/*v*) of 12N HCl or 0.072% (*v*/*v*) of 2 N NaOH (Sigma-Aldrich, Madrid, Spain), respectively. The competitive growth of the four *E. coli* strains and among the four *K. pneumoniae* strains was assessed in MHB, mixing by pairs 5 × 10^5^ CFU/mL each, in each experiment. At different time points, aliquots from the cultures were seeded on blood agar (Beckton Dickinson, Franklin Lakes, NJ, USA) and MH plates containing fosfomycin, ciprofloxacin, or amikacin to differentiate the growth of each of the four *E. coli* strains [45]. For the competition between *K. pneumoniae* strains, the same protocol described above was used, except in this case, the MH plates or filter-sterilized normal human urine contained ciprofloxacin or fosfomycin to differentiate between the *K. pneumoniae* strains [45]. A CI = 1 indicates that the fitness is equivalent; If the CI > 1, it indicates that the resistant strain is able to proliferate more than the susceptible strain; if CI < 1, the resistant strain is weaker than the susceptible strain [46].

#### 4.2.3. Biofilm Assay

Biofilm production was determined as previously described [47] with modifications to adjust the pH of MHB to acidic, neutral, or alkaline. The strains were cultured in MHB overnight at 37 °C and then diluted to 10^6^ CFU/mL using MHB. A total of 200 μL of the cell suspension was added to each well of a round-bottom 96-well plate and grown overnight at 37 °C. After incubation, each well was washed twice to remove non-adherent bacteria, and 200 μL of 0.4% crystal violet dye (Sigma-Aldrich, Madrid, Spain) was added to each well. Following 10 min at room temperature, the wells were washed twice, and 200 μL of 96% ethanol was added. After 15 min at room temperature, biofilm formation was quantified by measuring the optical density (OD) at 580 nm (Asys UVM 340 Microplate Reader, Cambridge, UK). MHB was used as negative control, and *E. coli* ATCC25922 and *K. pneumoniae* CECT997 were used as positive controls for *E. coli* and *K. pneumoniae* strains, respectively. The results were normalized to the neutral pH positive control strains, which were considered as 100%.

#### 4.2.4. Motility Assay

Surface motility was measured as previously described [48] with adjustments to obtain acidic, neutral, and alkaline media. Briefly, overnight cultures of each strain were adjusted in PBS to an OD 600 nm of 0.6 (Lonza, Verviers, Belgium). Three µL of the bacterial suspension was placed in a Luria-Bertani (LB) plate or in filter-sterilized normal human urine containing 0.3% agarose. Plates were incubated at 37 °C with 80% of humidity, and the diameter of surface extension was measured after 24 h of incubation.

#### 4.2.5. Adherence and Invasion to Human Cells

Bacterial adherence and invasion assays were performed as previously described [49]. Briefly, embryonic kidney epithelial cells, line HEK-293 (ATCC^®^ CRL-1573™), were seeded (10^5^ cells/well) for 24 h in 24-well plates. Strains were grown in MHB (Sigma-Aldrich, Madrid, Spain) at 37 °C for 20–24 h, washed with phosphate-buffered saline (PBS; Sigma-Aldrich, Madrid, Spain), and resuspended in DMEM (Biowest, Barcelona, Spain) prior to infecting the eukaryotic cell cultures. The cells were rinsed twice with PBS and incubated overnight with a 1:1000 dilution of cultures of all strains. The pH of DMEM was adjusted to acidic, neutral, or alkaline using HCl or NaOH. In addition, a growth control was added to the assay showing each bacterial strain adherence to or invasion of HEK-293 cells in DMEM without modification at pH 7.4.

### 4.3. In Vivo Studies

#### 4.3.1. Animals

Immunocompetent C57BL/6J female mice weighing 20 g (Production and Experimentation Animal Center, Seville, Spain) were used. The mice had murine pathogen-free sanitary status and were assessed for genetic authenticity. This study was conducted in accordance with the recommendations of the Guide for the Care and Use of Laboratory Animals [50]. Experiments were approved by the Committee on the Ethics of Animal Experiments of Consejería de Agricultura, Ganadería, Pesca y Desarrollo Sostenible, Spain (06/03/2018/022 and 16/11/2020/134).

#### 4.3.2. Characterization of UTI Murine Model at Different Urine pHs

A previously described UTI model was used [51]. Groups of six mice per inoculum were intraperitoneally anesthetized (ketamine/xylazine), and transurethral inoculation with 50 μL of bacterial dilutions of each strain starting from an inoculum of approximately 10^9^ CFU/mL and ending when the inoculum caused infection in the lower urinary tract of the animals. Then, the UTI model was characterized for the different strains at different urine pHs. Briefly, three days prior to inoculation and throughout the study, drinking water was replaced daily with (i) water containing 5% glucose (neutral urine pH group) or (ii) water containing 100 mM sucrose plus 0.56 M NH_4_Cl (acidic urine pH group) [52]. On the day of the inoculation, mice were intraperitoneally anaesthetized and transurethrally inoculated with 50 μL of the previously characterized doses: 9 log_10_ CFU/mL for *E. coli* Nu14 and *E. coli* HUVR94 and 8 log_10_ CFU/mL for the six remaining strains. Samples were drawn and processed immediately after death during this study or after euthanasia at the end of the study period (48 h post-inoculation). Blood samples were obtained for qualitative cultures, and urine and tissues (bladder and kidneys) were aseptically extracted for quantitative cultures [48].

To assess whether alkaline urine pH affected bacterial concentrations, experiments were performed in groups of three mice infected with *E. coli* Nu14 and *K. pneumoniae* HUVR42, with the same inoculum and follow-up as detailed above. To alkalinize the urine pH, drinking water was replaced daily with water containing 5% glucose and 0.5% NaHCO_3_ [53].

Histopathological studies were performed on the kidneys of randomly selected mice inoculated 48 h earlier with *E. coli* Nu14 and *K. pneumoniae* HUVR42 at three different urine pH levels. After euthanasia of the mice, kidneys were aseptically extracted, fixed (10% formaldehyde), and embedded in paraffin wax. Sections 4 μm thick were stained according to standard hematoxylin-eosin methods.

For immunocompromised animals, a protocol similar to that described above was used, except that these animals received 150 mg/kg and 100 mg/kg cyclophosphamide intraperitoneally on days −4 and −1 pre-inoculation (day 0), respectively [54]. The inoculum required to induce UTI was 1 log_10_ CFU/mL less than that in immunocompetent mice: 8 log_10_ CFU/mL for *E. coli* Nu14 and *E. coli* HUVR94 and 7 log_10_ CFU/mL for the remaining strains.

### 4.4. Statistical Analysis

Mortality and positive blood culture rates are expressed as percentages, and bacterial tissue and urine concentrations are presented as mean ± standard deviation of log_10_ CFU/g and log_10_ CFU/mL, respectively. Chi-square or Fisher’s exact tests were used to compare mortality and BSI between the groups. The Mann–Whitney U test was used to compare quantitative variables. *p* < 0.05 was considered statistically significant. Statistical analyses were conducted using SPSS v24.0 software (SPSS Inc., Chicago, IL, USA).

## Figures and Tables

**Table 1:** Strain minimum inhibitory concentration (MIC) of ciprofloxacin, fosfomycin, and amikacin and multi-locus sequence types (MLST) by Achtman [18] and Pasteur [19] scheme for *Escherichia coli* and *Klebsiella pneumoniae* strains, respectively.

	MIC CIP (mg/L)	MIC FOS (mg/L)	MIC AMK (mg/L)	MLST
*E. coli* Nu14	0.03	2	8	95
*E. coli* HUVR94	0.03	0.50	1	9744
*E. coli* Nu14 *gyrA* D87G	0.25	0.50	8	95
*E. coli* Nu14 *glpT* missense mutation	0.01	32	8	95
*K. pneumoniae* HUVR42	0.007	4	N/A	2701
*K. pneumoniae* HUVR5	8	128	N/A	466
*K. pneumoniae* HUVR110	8	4	N/A	342
*K. pneumoniae* HUVR91	64	0.06	N/A	91

CIP: ciprofloxacin; FOS: fosfomycin; AMK: amikacin; N/A: not performed.

**Table 2:** Bacterial concentrations in tissues, urine, and blood, and mortality rates in murine urinary tract infection by *Escherichia coli* strains, at acidic and neutral urine pH.

**Immunocompetent Mice Model**						
***E. coli* Strain**	**Urine pH**	**Kidneys** **(Log_10_ CFU/g)**	**Bladder** **(Log_10_ CFU/g)**	**Urine** **(Log_10_ CFU/mL)**	**BSI (%)**	**Mortality (%)**
Nu14	Acidic	3.66 ± 0.56	3.52 ± 0.43 ^a^	2.31 ± 2.44	20	20
	Neutral	1.28 ± 2.48	7.08 ± 0.03	6.02 ± 0.94	0	0
HUVR94	Acidic	4.91 ± 0.51 ^a^	4.90 ± 0.86	4.15 ± 0.79	20	0
	Neutral	2.88 ± 1.58	4.13 ± 0.76	3.40 ± 0.77	0	0
Nu14 *gyrA* D87G	Acidic	6.32 ± 1.21 ^a^	5.51 ± 1.86	5.45 ± 0.42	80	0
	Neutral	3.09 ± 1.01	5.19 ± 0.50	4.19 ± 2.39	0	0
Nu14 *glpT* missense mutation	Acidic	5.30 ± 1.47	4.93 ± 2.36	4.67 ± 1.63	40	20
	Neutral	2.59 ± 1.80	6.52 ± 1.67	5.93 ± 1.85	0	0
**Immunocompromised Mice Model**						
***E. coli* Strain**	**Urine pH**	**Kidneys** **(Log_10_ CFU/g)**	**Bladder** **(Log_10_ CFU/g)**	**Urine** **(Log_10_ CFU/mL)**	**BSI (%)**	**Mortality (%)**
Nu14	Acidic	6.25 ± 1.60 ^a^	4.23 ± 0.02	3.34 ± 1.04	50	0
	Neutral	0.62 ± 0.17	6.42 ± 0.17	6.75 ± 1.72	0	0
HUVR94	Acidic	7.80 ± 0.48 ^a^	1.21 ± 0.72	2.57 ± 2.23	33	0
	Neutral	0.98 ± 0.37	8.08 ± 0.66	8.31 ± 1.27	33	0
Nu14 *gyrA* D87G	Acidic	5.61 ± 1.45	3.77 ± 0.04	2.42 ± 3.43	100 ^a^	0
	Neutral	5.82 ± 0.20	7.20 ± 1.35	7.31 ± 1.54	17	0
Nu14 *glpT* missense mutation	Acidic	6.30 ± 1.31 ^a^	5.65 ± 0.19	2.68 ± 2.32	100 ^a^	0
	Neutral	2.46 ± 2.50	6.55 ± 1.98	8.38 ± 0.22	0	0

BSI: bloodstream infection; ^a^: *p* < 0.05 with respect to neutral urine pH.

**Table 3:** Bacterial concentrations in tissues and blood, and mortality rates in murine urinary tract infection by *Klebsiella pneumoniae* strains, at acidic and neutral urine pH.

**Immunocompetent Mice Model**						
***K. pneumoniae* Strain**	**Urine pH**	**Kidneys** **(Log_10_ CFU/g)**	**Bladder** **(Log_10_ CFU/g)**	**Urine** **(Log_10_ CFU/mL)**	**BSI (%)**	**Mortality (%)**
HUVR42	Acidic	4.48 ± 0.39 ^a^	2.50 ± 2.23 ^a^	6.60 ± 2.84	67 ^a^	0
	Neutral	1.36 ± 0.95	7.40 ± 1.03	6.68 ± 0.73	0	0
HUVR5	Acidic	3.98 ± 1.01 ^a^	4.22 ± 0.30 ^a^	4.30 ± 0.85 ^a^	0	0
	Neutral	0.29 ± 0.51	8.33 ± 0.32	8.55 ± 0.54	0	0
HUVR110	Acidic	4.19 ± 0.67 ^a^	4.39 ± 0.39 ^a^	4.51 ± 0.29 ^a^	0	0
	Neutral	0.00 ± 0.00	8.21 ± 0.31	8.70 ± 0.04	0	0
HUVR91	Acidic	7.80 ± 0.48 ^a^	1.21 ± 0.72 ^a^	2.57 ± 2.23 ^a^	67 ^a^	0
	Neutral	0.98 ± 0.37	8.08 ± 0.66	8.31 ± 1.27	0	0
**Immunocompromised Mice Model**						
***K. pneumoniae* Strain**	**Urine pH**	**Kidneys** **(Log_10_ CFU/g)**	**Bladder** **(Log_10_ CFU/g)**	**Urine** **(Log_10_ CFU/mL)**	**BSI (%)**	**Mortality (%)**
HUVR42	Acidic	4.61 ± 3.34 ^a^	5.56 ± 3.75	0.50 ± 1.00 ^a^	25	0
	Neutral	1.03 ± 2.07	6.02 ± 4.10	9.27 ± 0.21	0	0
HUVR5	Acidic	5.07 ± 3.46 ^a^	4.75 ± 2.99	3.88 ± 2.97	25	0
	Neutral	0.59 ± 0.67	2.86 ± 4.02	3.88 ± 3.88	0	0
HUVR110	Acidic	4.46 ± 1.85	3.46 ± 1.23	0.00 ± 0.00 ^a^	0	0
	Neutral	4.53 ± 0.49	5.26 ± 3.73	7.52 ± 1.96	0	0
HUVR91	Acidic	6.80 ± 1.47 ^a^	5.18 ± 1.42	6.27 ± 1.39	33	0
	Neutral	0.11 ± 0.13	2.94 ± 2.16	2.15 ± 2.70	0	0

BSI: bloodstream infection; ^a^: *p* < 0.05 with respect to neutral urine pH.

## Data Availability

The data presented in this study are available on request from the corresponding author.

## Supplementary Materials

The following supporting information can be downloaded at: https://www.mdpi.com/article/10.3390/ijms25147925/s1.

## References

[1] Ignatova N., Abidullina A., Streltsova O., Elagin V., Kamensky V. (2023). Effect of pH, Norepinephrine and Glucose on Metabolic and Biofilm Activity of Uropathogenic Microorganisms. Microorganisms.

[2] Ince B.A., Anderson E.J., Neer R.M. (2004). Lowering dietary protein to U.S. Recommended dietary allowance levels reduces urinary calcium excretion and bone resorption in young women. J. Clin. Endocrinol. Metab..

[3] Wu J., Gao Y. (2015). Physiological conditions can be reflected in human urine proteome and metabolome. Expert Rev. Proteom..

[4] Dobrek Ł. (2021). Drug-related urinary tract infections. Wiad. Lek..

[5] Barbara K., Amidou S. (2017). Virulence Factors and Innovative Strategies for the Treatment and Control of Uropathogenic *Escherichia coli*. Escherichia coli—Recent Advances on Physiology, Pathogenesis and Biotechnological Applications.

[6] Ronald A. (2003). The etiology of urinary tract infection: Traditional and emerging pathogens. Dis. Mon..

[7] Stamm W.E., McKevitt M., Roberts P.L., White N.J. (1991). Natural history of recurrent urinary tract infections in women. Rev. Infect. Dis..

[8] Vilas Boas D., Castro J., Araújo D., Nóbrega F.L., Keevil C.W., Azevedo N.F., Vieira M.J., Almeida C. (2024). The Role of Flagellum and Flagellum-Based Motility on *Salmonella Enteritidis* and *Escherichia coli* Biofilm Formation. Microorganisms.

[9] Connell I., Agace W., Klemm P., Schembri M., Mărild S., Svanborg C. (1996). Type 1 fimbrial expression enhances *Escherichia coli* virulence for the urinary tract. Proc. Natl. Acad. Sci. USA.

[10] Nowicki B., Selvarangan R., Nowicki S. (2001). Family of *Escherichia coli* Dr adhesins: Decay-accelerating factor receptor recognition and invasiveness. J. Infect. Dis..

[11] Spurbeck R.R., Mobley H.L.T., Donnenberg M. (2013). Chapter 9—Uropathogenic *Escherichia coli*. Escherichia coli.

[12] Ribić R., Meštrović T., Neuberg M., Kozina G. (2018). Effective anti-adhesives of uropathogenic *Escherichia coli*. Acta Pharm..

[13] Alcántar-Curiel M.D., Blackburn D., Saldaña Z., Gayosso-Vázquez C., Iovine N.M., De la Cruz M.A., Girón J.A. (2013). Multi-functional analysis of *Klebsiella pneumoniae* fimbrial types in adherence and biofilm formation. Virulence.

[14] Govindarajan D.K., Kandaswamy K. (2022). Virulence factors of uropathogens and their role in host pathogen interactions. Cell Surf..

[15] Herrera-Espejo S., Fontserè S., Infante C., Suárez-Benjumea A., Carretero-Ledesma M., Suñer-Poblet M., González-Corvillo C., Bernal G., Martín-Gutiérrez G., Pérez-Cáceres J.A. (2024). Acidic Urine pH and Clinical Outcome of Lower Urinary Tract Infection in Kidney Transplant Recipients Treated with Ciprofloxacin and Fosfomycin. Antibiotics.

[16] Harjai K., Khandwahaa R.K., Mittal R., Yadav V., Gupta V., Sharma S. (2005). Effect of pH on production of virulence factors by biofilm cells of *Pseudomonas aeruginosa*. Folia Microbiol..

[17] Klinth J.E., Castelain M., Uhlin B.E., Axner O. (2012). The influence of pH on the specific adhesion of P piliated *Escherichia coli*. PLoS ONE.

[18] Wirth T., Falush D., Lan R., Colles F., Mensa P., Wieler L.H., Karch H., Reeves P.R., Maiden M.C., Ochman H. (2006). Sex and virulence in *Escherichia coli*: An evolutionary perspective. Mol. Microbiol..

[19] Diancourt L., Passet V., Verhoef J., Grimont P.A., Brisse S. (2005). Multilocus sequence typing of *Klebsiella pneumoniae* nosocomial isolates. J. Clin. Microbiol..

[20] Tannenbaum J., Bennett B.T. (2015). Russell and Burch’s 3Rs then and now: The need for clarity in definition and purpose. J. Am. Assoc. Lab. Anim. Sci..

[21] Martín-Gutiérrez G., Docobo-Pérez F., Rodriguez-Beltrán J., Rodríguez-Martínez J.M., Aznar J., Pascual A., Blázquez J. (2018). Urinary Tract Conditions Affect Fosfomycin Activity against *Escherichia coli* Strains Harboring Chromosomal Mutations Involved in Fosfomycin Uptake. Antimicrob. Agents Chemother..

[22] Beerepoot M., Geerlings S. (2016). Non-Antibiotic Prophylaxis for Urinary Tract Infections. Pathogens.

[23] Carlsson S., Govoni M., Wiklund N.P., Weitzberg E., Lundberg J.O. (2003). In vitro evaluation of a new treatment for urinary tract infections caused by nitrate-reducing bacteria. Antimicrob. Agents Chemother..

[24] Peng H., Purkerson J.M., Freeman R.S., Schwaderer A.L., Schwartz G.J. (2020). Acidosis induces antimicrobial peptide expression and resistance to uropathogenic *E. coli* infection in kidney collecting duct cells via HIF-1α. Am. J. Physiol. Renal Physiol..

[25] Hains D.S., Chen X., Saxena V., Barr-Beare E., Flemming W., Easterling R., Becknell B., Schwartz G.J., Schwaderer A.L. (2014). Carbonic anhydrase 2 deficiency leads to increased pyelonephritis susceptibility. Am. J. Physiol. Renal Physiol..

[26] Purkerson J.M., Corley J.L., Schwartz G.J. (2020). Metabolic acidosis exacerbates pyelonephritis in mice prone to vesicoureteral reflux. Physiol. Rep..

[27] Shields-Cutler R.R., Crowley J.R., Hung C.S., Stapleton A.E., Aldrich C.C., Marschall J., Henderson J.P. (2015). Human Urinary Composition Controls Antibacterial Activity of Siderocalin. J. Biol. Chem..

[28] Zhou Y., Cheng Y., Ma T., Wang J., Li S., Wang J., Han L., Hou X., Ma X., Jiang S. (2023). Transcriptomic and phenotype analysis revealed the role of *rpoS* in stress resistance and virulence of a novel ST3355 ESBL-producing hypervirulent *Klebsiella pneumoniae* isolate. Front. Cell Infect. Microbiol..

[29] Alav I., Sutton J.M., Rahman K.M. (2018). Role of bacterial efflux pumps in biofilm formation. J. Antimicrob. Chemother..

[30] Song X., Hou M., Tu J., Xue M., Shao Y., Jiang H., Liu H., Xue T., Wang G., Qi K. (2019). Outer membrane proteins YbjX and PagP co-regulate motility in *Escherichia coli* via the bacterial chemotaxis pathway. Res. Vet. Sci..

[31] Tarlton N.J., Moritz C., Adams-Sapper S., Riley L.W. (2019). Genotypic analysis of uropathogenic *Escherichia coli* to understand factors that impact the prevalence of β-lactam-resistant urinary tract infections in a community. J. Glob. Antimicrob. Resist..

[32] Riley L.W. (2014). Pandemic lineages of extraintestinal pathogenic *Escherichia coli*. Clin. Microbiol. Infect..

[33] Yang J., Ye L., Guo L., Zhao Q., Chen R., Luo Y., Chen Y., Tian S., Zhao J., Shen D. (2013). A nosocomial outbreak of KPC-2-producing *Klebsiella pneumoniae* in a Chinese hospital: Dissemination of ST11 and emergence of ST37, ST392 and ST395. Clin. Microbiol. Infect..

[34] Ballén V., Sáez E., Benmessaoud R., Houssain T., Alami H., Barkat A., Kabiri M., Moraleda C., Bezad R., Vila J. (2015). First report of a *Klebsiella pneumoniae* ST466 strain causing neonatal sepsis harbouring the blaCTX-M-15 gene in Rabat, Morocco. FEMS Microbiol. Lett..

[35] Grünzweil O.M., Palmer L., Cabal A., Szostak M.P., Ruppitsch W., Kornschober C., Korus M., Misic D., Bernreiter-Hofer T., Korath A.D.J. (2021). Presence of β-Lactamase-producing Enterobacterales and Salmonella Isolates in Marine Mammals. Int. J. Mol. Sci..

[36] Ong C.L., Ulett G.C., Mabbett A.N., Beatson S.A., Webb R.I., Monaghan W., Nimmo G.R., Looke D.F., McEwan A.G., Schembri M.A. (2008). Identification of type 3 fimbriae in uropathogenic *Escherichia coli* reveals a role in biofilm formation. J. Bacteriol..

[37] Soto S. (2014). Importance of Biofilms in Urinary Tract Infections: New Therapeutic Approaches. Adv. Biol..

[38] Welin-Neilands J., Svensäter G. (2007). Acid tolerance of biofilm cells of *Streptococcus mutans*. Appl. Environ. Microbiol..

[39] Valdebenito M., Crumbliss A.L., Winkelmann G., Hantke K. (2006). Environmental factors influence the production of enterobactin, salmochelin, aerobactin, and yersiniabactin in *Escherichia coli* strain Nissle 1917. Int. J. Med. Microbiol..

[40] van Raaij S.E.G., Rennings A.J., Biemond B.J., Schols S.E.M., Wiegerinck E.T.G., Roelofs H.M.J., Hoorn E.J., Walsh S.B., Nijenhuis T., Swinkels D.W. (2019). Iron handling by the human kidney: Glomerular filtration and tubular reabsorption both contribute to urinary iron excretion. Am. J. Physiol. Renal Physiol..

[41] Mehershahi K.S., Chen S.L. (2017). Complete Genome Sequence of the Uropathogenic *Escherichia coli* Strain NU14. Genome Announc..

[42] Komp Lindgren P., Marcusson L.L., Sandvang D., Frimodt-Møller N., Hughes D. (2005). Biological cost of single and multiple norfloxacin resistance mutations in *Escherichia coli* implicated in urinary tract infections. Antimicrob. Agents Chemother..

[43] Nilsson A.I., Berg O.G., Aspevall O., Kahlmeter G., Andersson D.I. (2003). Biological costs and mechanisms of fosfomycin resistance in *Escherichia coli*. Antimicrob. Agents Chemother..

[44] Larsen M.V., Cosentino S., Rasmussen S., Friis C., Hasman H., Marvig R.L., Jelsbak L., Sicheritz-Pontén T., Ussery D.W., Aarestrup F.M. (2012). Multilocus sequence typing of total-genome-sequenced bacteria. J. Clin. Microbiol..

[45] Labrador-Herrera G., Pérez-Pulido A.J., Álvarez-Marín R., Casimiro-Soriguer C.S., Cebrero-Cangueiro T., Morán-Barrio J., Pachón J., Viale A.M., Pachón-Ibáñez M.E. (2020). Virulence role of the outer membrane protein CarO in carbapenem-resistant *Acinetobacter baumannii*. Virulence.

[46] Auerbuch V., Lenz L.L., Portnoy D.A. (2001). Development of a competitive index assay to evaluate the virulence of *Listeria monocytogenes actA* mutants during primary and secondary infection of mice. Infect. Immun..

[47] Recacha E., Machuca J., Díaz-Díaz S., García-Duque A., Ramos-Guelfo M., Docobo-Pérez F., Blázquez J., Pascual A., Rodríguez-Martínez J.M. (2019). Suppression of the SOS response modifies spatiotemporal evolution, post-antibiotic effect, bacterial fitness and biofilm formation in quinolone-resistant *Escherichia coli*. J. Antimicrob. Chemother..

[48] Cebrero-Cangueiro T., Labrador-Herrera G., Carretero-Ledesma M., Herrera-Espejo S., Álvarez-Marín R., Pachón J., Cisneros J.M., Pachón-Ibáñez M.E. (2022). IgM-enriched immunoglobulin improves colistin efficacy in a pneumonia model by *Pseudomonas aeruginosa*. Life Sci. Alliance.

[49] Gil-Marqués M.L., Labrador Herrera G., Miró Canturri A., Pachón J., Smani Y., Pachón-Ibáñez M.E. (2020). Role of PstS in the Pathogenesis of *Acinetobacter baumannii* Under Microaerobiosis and Normoxia. J. Infect. Dis..

[50] NRC (National Research Council) (2011). Guide for the Care and Use of Laboratory Animals.

[51] Moazzezy N., Asadi Karam M.R., Rafati S., Bouzari S., Oloomi M. (2020). A Synthetic Peptide 2Abz(23)S(29) Reduces Bacterial Titer and Induces Pro-Inflammatory Cytokines in a Murine Model of Urinary Tract Infection. Drug Des. Dev. Ther..

[52] Vasandani V.M., Burris J.A., Sung C. (1999). Reversible nephrotoxicity of onconase and effect of lysine pH on renal onconase uptake. Cancer Chemother. Pharmacol..

[53] Robey I.F., Nesbit L.A. (2013). Investigating mechanisms of alkalinization for reducing primary breast tumor invasion. Biomed. Res. Int..

[54] Docobo-Pérez F., López-Rojas R., Domínguez-Herrera J., Jiménez-Mejias M.E., Pichardo C., Ibáñez-Martínez J., Pachón J. (2012). Efficacy of linezolid versus a pharmacodynamically optimized vancomycin therapy in an experimental pneumonia model caused by methicillin-resistant *Staphylococcus aureus*. J. Antimicrob. Chemother..

