# Supplementary material for: Effects of pH on the Pathogenicity of Escherichia coli and Klebsiella pneumoniae on the Kidney: In Vitro and In Vivo Studies
Source: Int J Mol Sci. 2024 Jul 19;25(14):7925. doi: 10.3390/ijms25147925 (PMC11277208; doi:10.3390/ijms25147925)
Supplement: Supplementary file 1 [file ijms-25-07925-s001.zip › ijms-3076093-supplementary.pdf]

Supplementary Materials

Table S1. Bacterial concentration in tissues and urine, at neutral and alkaline urine pH, in mice after 48 hours of transurethral route infection with *E. coli* Nu14 and *K. pneumoniae* HUVR42 strains in immunocompetent and immunosuppressed mice model.

Bacterial Strain	pH Urine Condition	Kidneys (Log_{10} CFU/g)	Bladder (Log_{10} CFU/g)	Urine (Log_{10} CFU/mL)	BSI (%)	Mortality (%)
E. coli Nu14 (IC)	Alkaline	0.64±1.28	5.84±1.18	5.64±2.19	0	0
	Neutral	1.28±2.48	7.08±0.03	6.02±0.94	0	0
K. pneumoniae HUVR42 (IC)	Alkaline	2.75±0.10	6.98±0.98	5.50±2.54	0	0
	Neutral	1.36±0.95	7.40±1.03	6.68±0.73	0	0
E. coli Nu14 (ID)	Alkaline	4.25±0.13	5.71±0.92	8.01±0.11	0	0
	Neutral	0.62±0.17	6.42±0.17	6.75±1.72	0	0
K. pneumoniae HUVR42 (ID)	Alkaline	2.07±1.79	0.92±2.24	0.00±0.00[a]	0	0
	Neutral	1.03±2.07	6.02±4.10	9.27±0.21	0	0

IC: Immunocompetent; ID: Immunocompromised; [a]: $P = 0.057$ respect to neutral pH.

Table S2. Primers of housekeeping genes to multi-locus sequence typing (MLST) of *Escherichia coli* and *Klebsiella pneumoniae* strains.

E. coli Strains		*K. pneumoniae* Strains	
**Gene**	**Sequence (5′- 3′)**	**Gene**	**Sequence (5′- 3′)**
adk	F – ATTCTGCTTGGCGCTCCGGG<br>R – CCGTCAACTTTCGCGTATTT	*gapA*	F – GTTTTCCCAGTCACGACGTTGTATGAAATATGACTCCACTCACGG<br>R – TTGTGAGCGGATAACAATTTCCTTCAGAAGCGGCTTTGATGGCTT
fumC	F – CACAGGTCGCCAGCGCTTC<br>R – GTACGCAGCGAAAAAGATTC	*rpoB*	F – GTTTTCCCAGTCACGACGTTGTAGGCGAAATGGCWGAGAACCA<br>R – TTGTGAGCGGATAACAATTTCGAGTCTTCGAAGTTGTAACC
gyrB	F – TCGGCGACACGGATGACGGC<br>R – GTCCATGTAGGCGTTCAGGG	*phoE*	F – GTTTTCCCAGTCACGACGTTGTAACCTACCGCAACACCCAGTTCTTCGG<br>R – TTGTGAGCGGATAACAATTTCTGATCAGAACTGGTAGGTGAT
icd	F – TGGAAAGTAAAGTAGTTGTTCCGGCACA<br>R – GGACGCAGCAGGATCTGTT	*mdh*	F – GTTTTCCCAGTCACGACGTTGTACCCAACTCGCTTCAGGTTCAG<br>R – TTGTGAGCGGATAACAATTTCCCGTTTTTCCCCAGCAGCAG
mdh	F – TGAAAGTCGCAGTCCTCGGCGCTGCTGGCGG	*infB*	F – GTTTTCCCAGTCACGACGTTGTACTCGCTGCTGGACTATATTCG

	R – TTAACGAACTCCTGCCCCAGAGCGATATCTTTCTT		R – TTGTGAGCGGATAACAATTTCCGCTTTCAGCTCAAGACTTC
purA	F – CGCGCTGATGAAAGAGATGA<br>R – CATACGGTAAGCCACGCAGA	*tonB*	F – GTTTTCCCAGTCACGACGTTGTACTTTATACCTCGGTACATCAGGTT<br>R – TTGTGAGCGGATAACAATTTCATTCGCCGGCTGRGCRGAGAG
recA	F – CGCATTCGCTTTACCCTGACC<br>R – TCGTCGAAATCTACGGACCGGA	*pgi*	F – GAGAAAAACCTGCCTGTACTGCTGGC<br>R – CGCGCCACGCTTTATAGCGGTTAAT

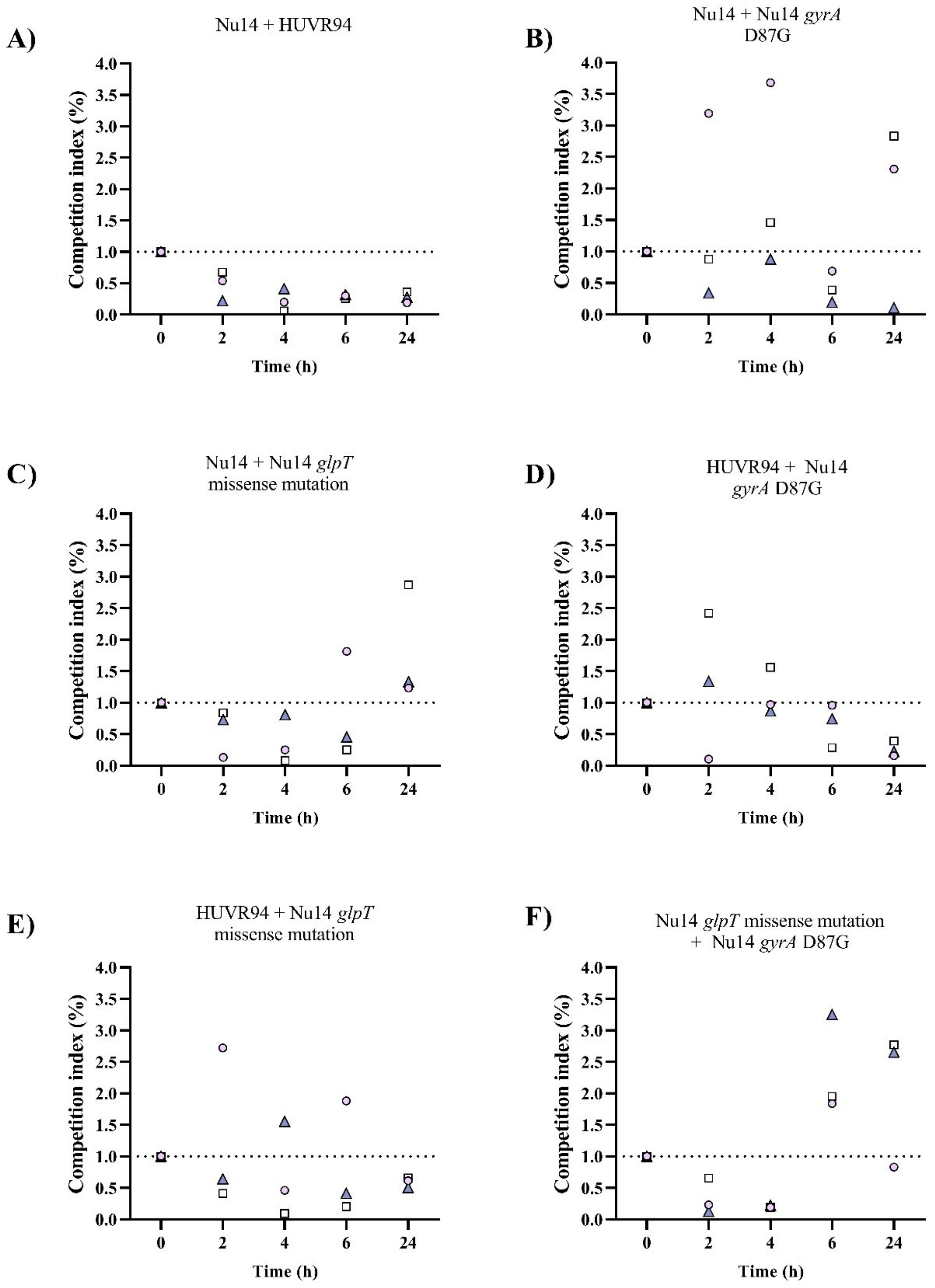

Figure S1. Competition growth curves in Müller-Hinton of the *Escherichia coli* Nu14, HUVR94 clinical isolate, Nu14 *gyrA* D87G and Nu14 *glpT* missense mutation strains at different pH. (**A**) Competition between *E. coli* Nu14 (amikacin MIC: 8 mg/L) and *E. coli* HUVR94 (amikacin MIC: 1 mg/L), seeded in blood agar plates and agar plates supplemented with amikacin at 4 mg/L; (**B**) Competition between *E. coli* Nu14 (ciprofloxacin MIC: 0.03 mg/L) and *E. coli* Nu14 *gyrA* (D87G) (ciprofloxacin MIC: 0.25 mg/L), seeded in blood agar plates and agar plates supplemented with

ciprofloxacin at 0.12 mg/L; (**C**) Competition between *E. coli* Nu14 (fosfomycin MIC: 2 mg/L) and *E. coli* Nu14 with *glpT* missense mutation (fosfomycin MIC: 32 mg/L), seeded in blood agar plates and agar plates supplemented with fosfomycin at 16 mg/L; (**D**) Competition between *E. coli* HUVR94 (amikacin MIC: 1 mg/L) and *E. coli* Nu14 *gyrA* (D87G) (amikacin MIC: 8 mg/L), seeded in blood agar plates and agar plates supplemented with amikacin at 4 mg/L; (**E**) Competition between *E. coli* HUVR94 (amikacin MIC: 1 mg/L) and *E. coli* Nu14 with *glpT* missense mutation (amikacin MIC: 8 mg/L), seeded in blood agar plates and agar plates supplemented with amikacin at 4 mg/L ; (**F**) Competition between E. coli Nu14 *gyrA* (D87G) (fosfomycin MIC: 0.50 mg/L) and *E. coli* Nu14 with *glpT* missense mutation (fosfomycin MIC: 32 mg/L), seeded in blood agar plates and agar plates supplemented with fosfomycin at 16 mg/L; Pointed bar: Competition index (CI) = 1; CI > 1, the strain growing in the agar plate supplemented with antibiotic is able to proliferate more than the strain with a lower MIC; CI < 1, the strain growing in the agar plate supplemented with antibiotic is weaker than the strain with a lower MIC; Pink circle: Acidic pH; White square: Neutral pH; Purple triangle: Alkaline pH.

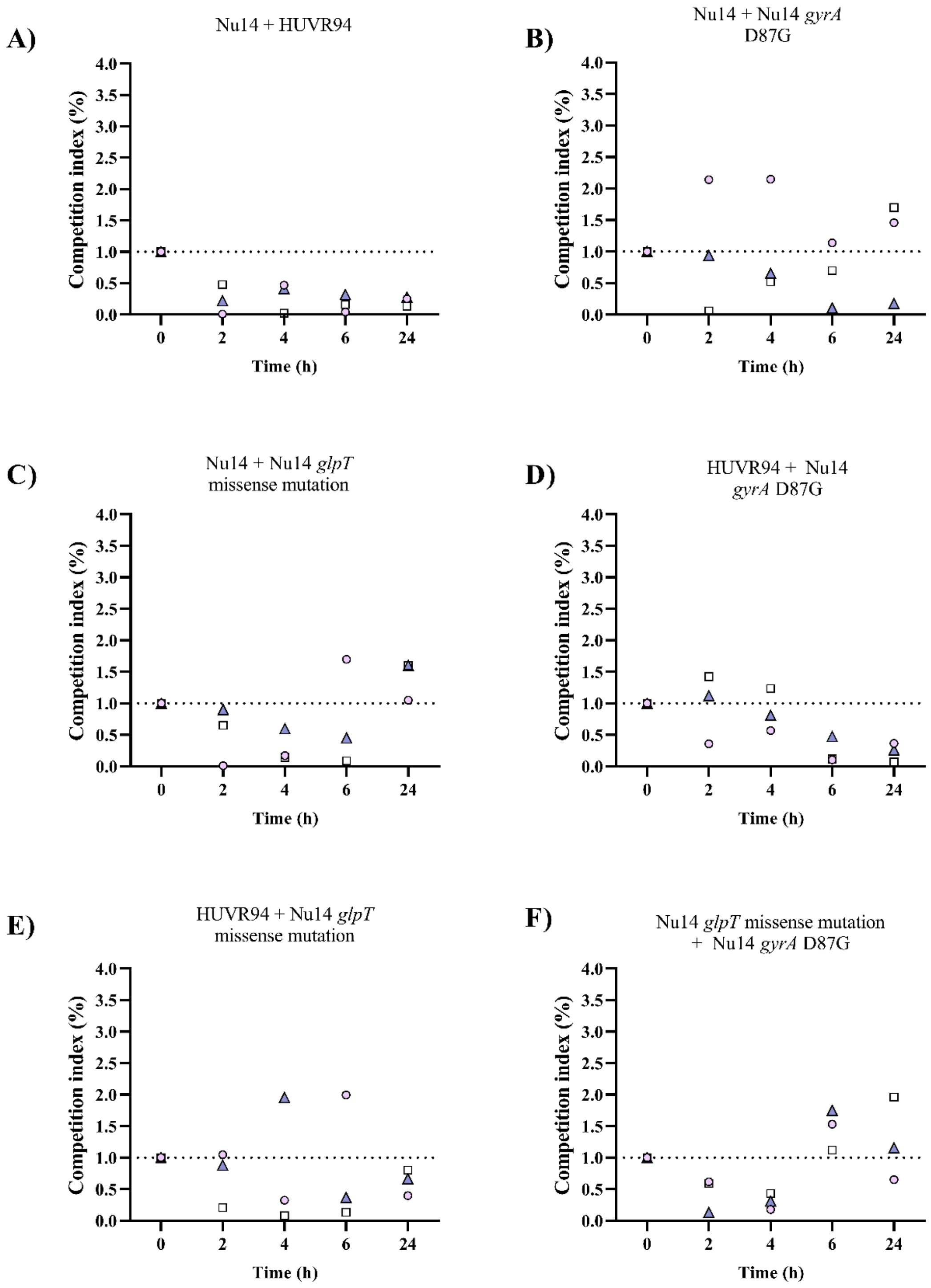

Figure S2. Competition growth curves in filter-sterilized human urine of the *Escherichia coli* Nu14, HUVR94 clinical isolate, Nu14 *gyrA* D87G and Nu14 *glpT* missense mutation strains at different pH. (**A**) Competition between *E. coli* Nu14 (amikacin MIC: 8 mg/L) and *E. coli* HUVR94 (amikacin MIC: 1 mg/L), seeded in blood agar plates and agar plates supplemented with amikacin at 4 mg/L; (**B**) Competition between *E. coli* Nu14 (ciprofloxacin MIC: 0.03 mg/L) and *E. coli* Nu14 *gyrA*

(D87G) (ciprofloxacin MIC: 0.25 mg/L), seeded in blood agar plates and agar plates supplemented with ciprofloxacin at 0.12 mg/L; (**C**) Competition between *E. coli* Nu14 (fosfomycin MIC: 2 mg/L) and *E. coli* Nu14 with *glpT* missense mutation (fosfomycin MIC: 32 mg/L), seeded in blood agar plates and agar plates supplemented with fosfomycin at 16 mg/L; (**D**) Competition between *E. coli* HUVR94 (amikacin MIC: 1 mg/L) and *E. coli* Nu14 *gyrA* (D87G) (amikacin MIC: 8 mg/L), seeded in blood agar plates and agar plates supplemented with amikacin at 4 mg/L; (**E**) Competition between *E. coli* HUVR94 (amikacin MIC: 1 mg/L) and *E. coli* Nu14 with *glpT* missense mutation (amikacin MIC: 8 mg/L), seeded in blood agar plates and agar plates supplemented with amikacin at 4 mg/L ; (**F**) Competition between E. coli Nu14 *gyrA* (D87G) (fosfomycin MIC: 0.50 mg/L) and *E. coli* Nu14 with *glpT* missense mutation (fosfomycin MIC: 32 mg/L), seeded in blood agar plates and agar plates supplemented with fosfomycin at 16 mg/L; Pointed bar: Competition index (CI) = 1; CI > 1, the strain growing in the agar plate supplemented with antibiotic is able to proliferate more than the strain with a lower MIC; CI < 1, the strain growing in the agar plate supplemented with antibiotic is weaker than the strain with a lower MIC; Pink circle: Acidic pH; White square: Neutral pH; Purple triangle: Alkaline pH.

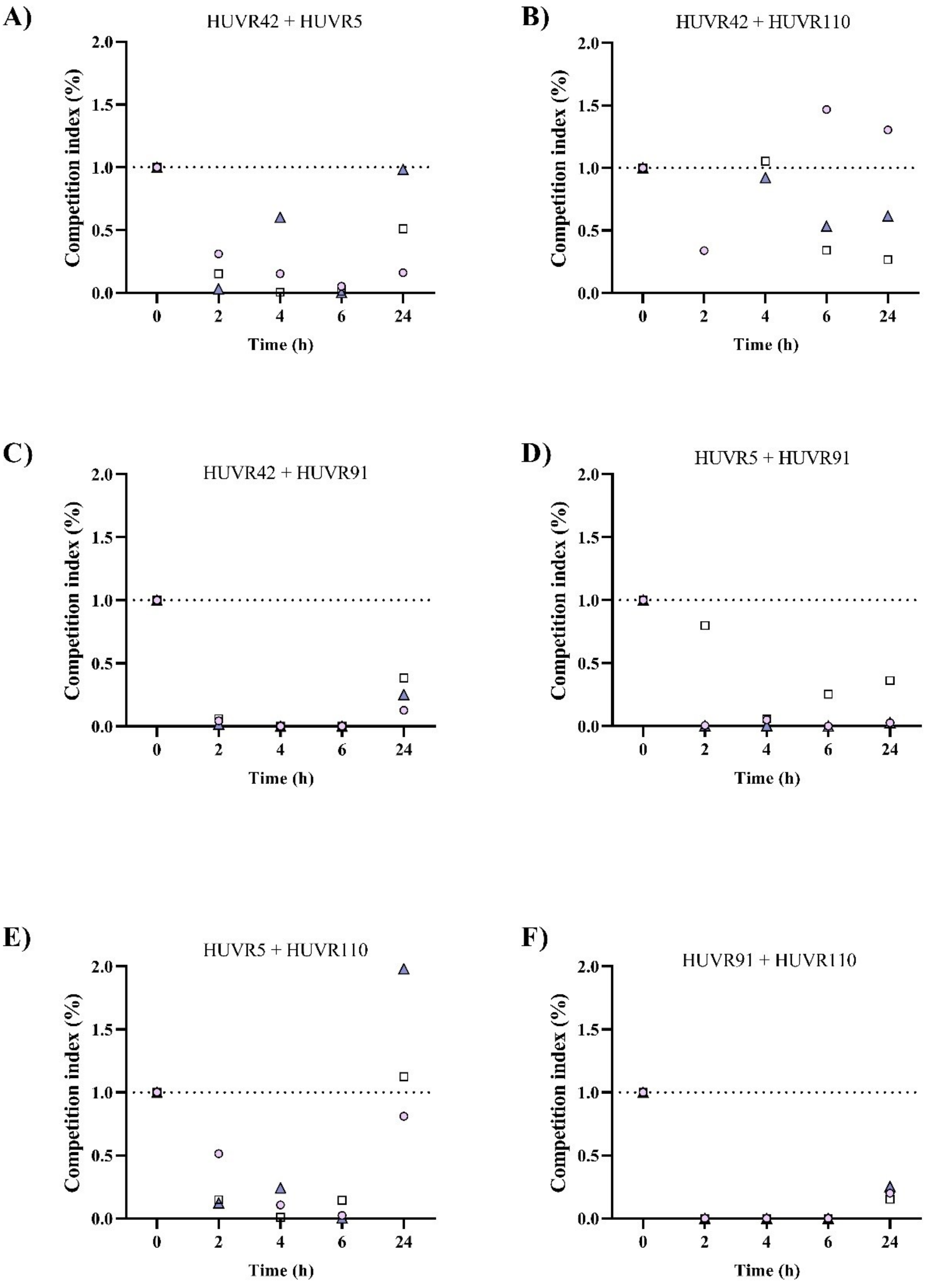

Figure S3. Competition growth curves in Müller-Hinton of the *Klebsiella pneumoniae* HUVR42, HUVR5, HUVR91 and HUVR110 strains at different pH. (**A**) Competition between *K. pneumoniae* HUVR42 (fosfomycin MIC: 4 mg/L) and *K. pneumoniae* HUVR5 (fosfomycin MIC: 128 mg/L), seeded in blood agar plates and agar plates supplemented with fosfomycin at 64 mg/L; (**B**) Competition between *K. pneumoniae* HUVR42 (ciprofloxacin MIC: 0.007 mg/L) and *K. pneumoniae*

HUVR110 (ciprofloxacin MIC: 8 mg/L), seeded in blood agar plates and agar plates supplemented with ciprofloxacin at 4 mg/L; (**C**) Competition between *K. pneumoniae* HUVR42 (ciprofloxacin MIC: 0.007 mg/L) and *K. pneumoniae* HUVR91 (ciprofloxacin MIC: 64 mg/L), seeded in blood agar plates and agar plates supplemented with ciprofloxacin at 32 mg/L; (**D**) Competition between *K. pneumoniae* HUVR5 (fosfomycin MIC: 128 mg/L) and *K. pneumoniae* HUVR110 (fosfomycin MIC: 4 mg/L), seeded in blood agar plates and agar plates supplemented with fosfomycin at 64 mg/L; (**E**) Competition between *K. pneumoniae* HUVR5 (fosfomycin MIC: 128 mg/L) and *K. pneumoniae* HUVR91 (fosfomycin MIC: 0.06 mg/L), seeded in blood agar plates and agar plates supplemented with fosfomycin at 64 mg/L; (**F**) Competition between *K. pneumoniae* HUVR110 (ciprofloxacin MIC: 8 mg/L) and *K. pneumoniae* HUVR91 (ciprofloxacin MIC: 64 mg/L), seeded in blood agar plates and agar plates supplemented with ciprofloxacin at 32 mg/L; Pointed bar: CI = 1; CI > 1, the strain growing in the agar plate supplemented with antibiotic is able to proliferate more than the strain with a lower MIC; CI < 1, the strain growing in the agar plate supplemented with antibiotic is weaker than the strain with a lower MIC; Pink circle: Acidic pH; White square: Neutral pH; Purple triangle: Alkaline pH.

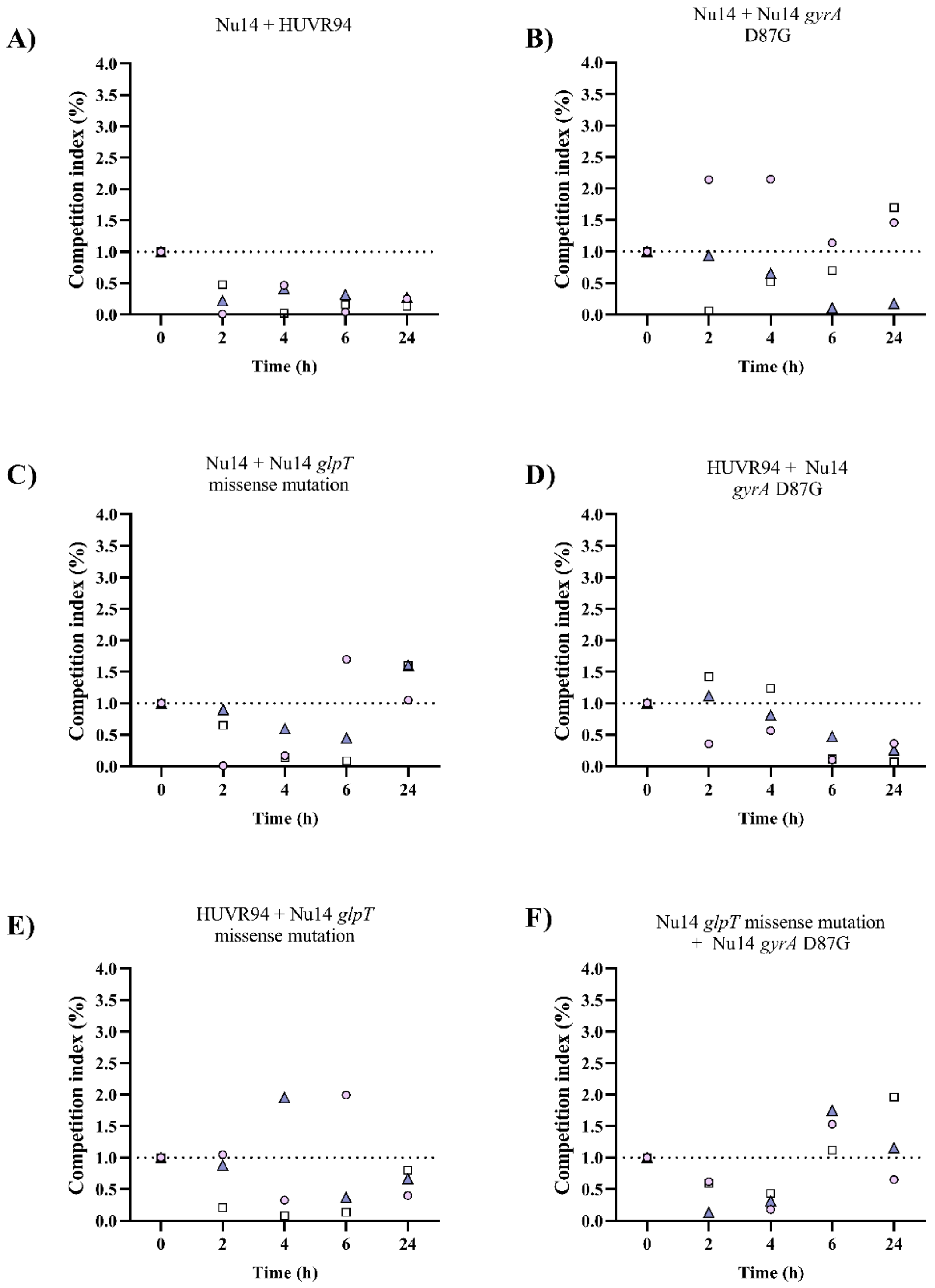

Figure S3. Competition growth curves in filter-sterilized human urine of the *Escherichia coli* Nu14, HUVR94 clinical isolate, Nu14 *gyrA* D87G and Nu14 *glpT* missense mutation strains at different pH. (**A**) Competition between *E. coli* Nu14 (amikacin MIC: 8 mg/L) and *E. coli* HUVR94 (amikacin MIC: 1 mg/L), seeded in blood agar plates and agar plates supplemented with amikacin at 4 mg/L; (**B**) Competition between *E. coli* Nu14 (ciprofloxacin MIC: 0.03 mg/L) and *E. coli* Nu14 *gyrA*

(D87G) (ciprofloxacin MIC: 0.25 mg/L), seeded in blood agar plates and agar plates supplemented with ciprofloxacin at 0.12 mg/L; (**C**) Competition between *E. coli* Nu14 (fosfomycin MIC: 2 mg/L) and *E. coli* Nu14 with *glpT* missense mutation (fosfomycin MIC: 32 mg/L), seeded in blood agar plates and agar plates supplemented with fosfomycin at 16 mg/L; (**D**) Competition between *E. coli* HUVR94 (amikacin MIC: 1 mg/L) and *E. coli* Nu14 *gyrA* (D87G) (amikacin MIC: 8 mg/L), seeded in blood agar plates and agar plates supplemented with amikacin at 4 mg/L; (**E**) Competition between *E. coli* HUVR94 (amikacin MIC: 1 mg/L) and *E. coli* Nu14 with *glpT* missense mutation (amikacin MIC: 8 mg/L), seeded in blood agar plates and agar plates supplemented with amikacin at 4 mg/L ; (**F**) Competition between E. coli Nu14 *gyrA* (D87G) (fosfomycin MIC: 0.50 mg/L) and *E. coli* Nu14 with *glpT* missense mutation (fosfomycin MIC: 32 mg/L), seeded in blood agar plates and agar plates supplemented with fosfomycin at 16 mg/L; Pointed bar: Competition index (CI) = 1; CI > 1, the strain growing in the agar plate supplemented with antibiotic is able to proliferate more than the strain with a lower MIC; CI < 1, the strain growing in the agar plate supplemented with antibiotic is weaker than the strain with a lower MIC; Pink circle: Acidic pH; White square: Neutral pH; Purple triangle: Alkaline pH.

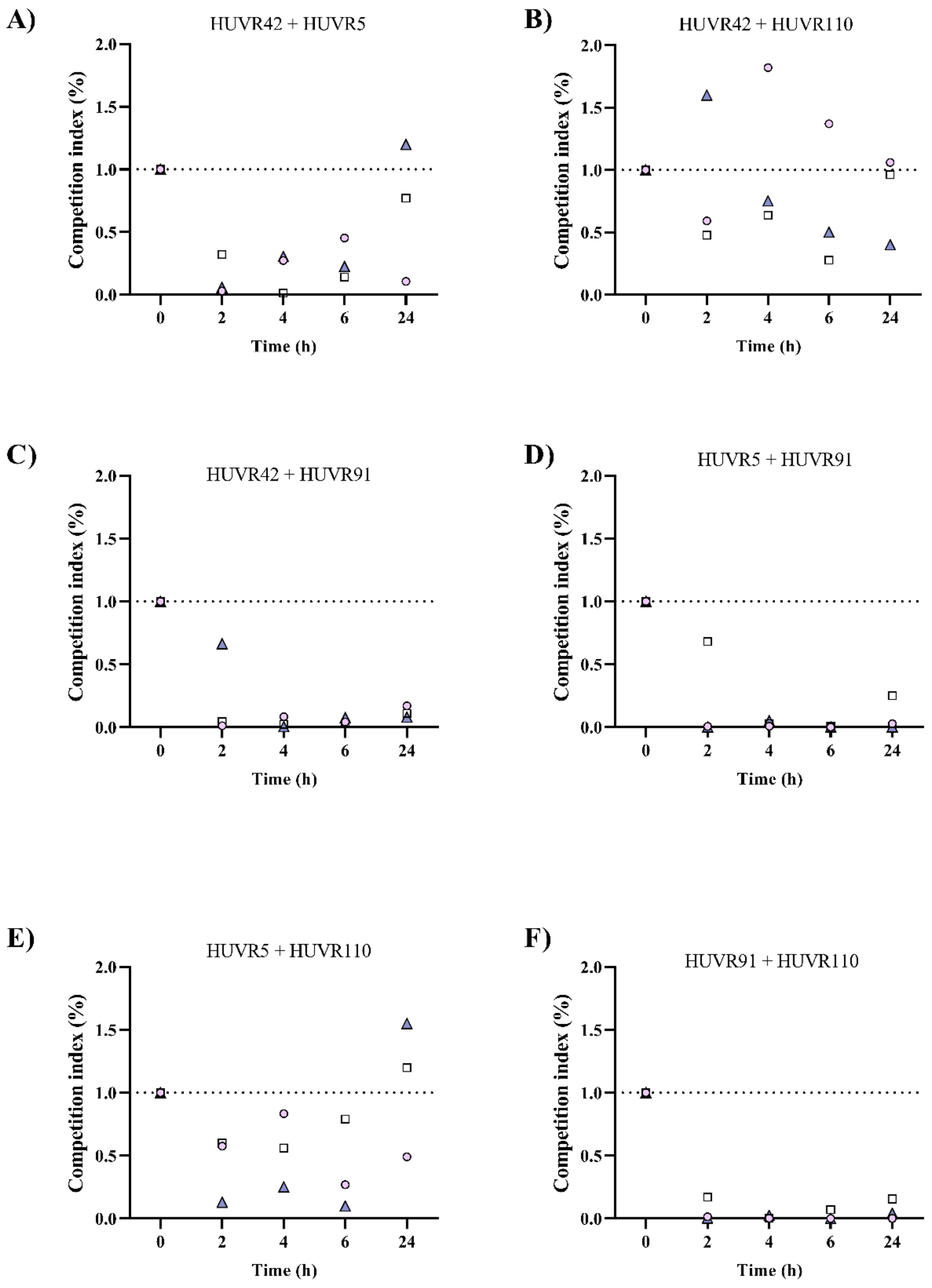

Figure S4. Competition growth curves in filter-sterilized human urine of the *Klebsiella pneumoniae* HUVR42, HUVR5, HUVR91 and HUVR110 strains at different pH. (**A**) Competition between *K. pneumoniae* HUVR42 (fosfomycin MIC: 4 mg/L) and *K. pneumoniae* HUVR5 (fosfomycin MIC: 128 mg/L), seeded in blood agar plates and agar plates supplemented with fosfomycin at 64 mg/L;

(**B**) Competition between *K. pneumoniae* HUVR42 (ciprofloxacin MIC: 0.007 mg/L) and *K. pneumoniae* HUVR110 (ciprofloxacin MIC: 8 mg/L), seeded in blood agar plates and agar plates supplemented with ciprofloxacin at 4 mg/L; (**C**) Competition between *K. pneumoniae* HUVR42 (ciprofloxacin MIC: 0.007 mg/L) and *K. pneumoniae* HUVR91 (ciprofloxacin MIC: 64 mg/L), seeded in blood agar plates and agar plates supplemented with ciprofloxacin at 32 mg/L; (**D**) Competition between *K. pneumoniae* HUVR5 (fosfomycin MIC: 128 mg/L) and *K. pneumoniae* HUVR110 (fosfomycin MIC: 4 mg/L), seeded in blood agar plates and agar plates supplemented with fosfomycin at 64 mg/L; (**E**) Competition between *K. pneumoniae* HUVR5 (fosfomycin MIC: 128 mg/L) and *K. pneumoniae* HUVR91 (fosfomycin MIC: 0.06 mg/L), seeded in blood agar plates and agar plates supplemented with fosfomycin at 64 mg/L; (**F**) Competition between *K. pneumoniae* HUVR110 (ciprofloxacin MIC: 8 mg/L) and *K. pneumoniae* HUVR91 (ciprofloxacin MIC: 64 mg/L), seeded in blood agar plates and agar plates supplemented with ciprofloxacin at 32 mg/L; Pointed bar: CI = 1; CI > 1, the strain growing in the agar plate supplemented with antibiotic is able to proliferate more than the strain with a lower MIC; CI < 1, the strain growing in the agar plate supplemented with antibiotic is weaker than the strain with a lower MIC; Pink circle: Acidic pH; White square: Neutral pH; Purple triangle: Alkaline pH.

A)

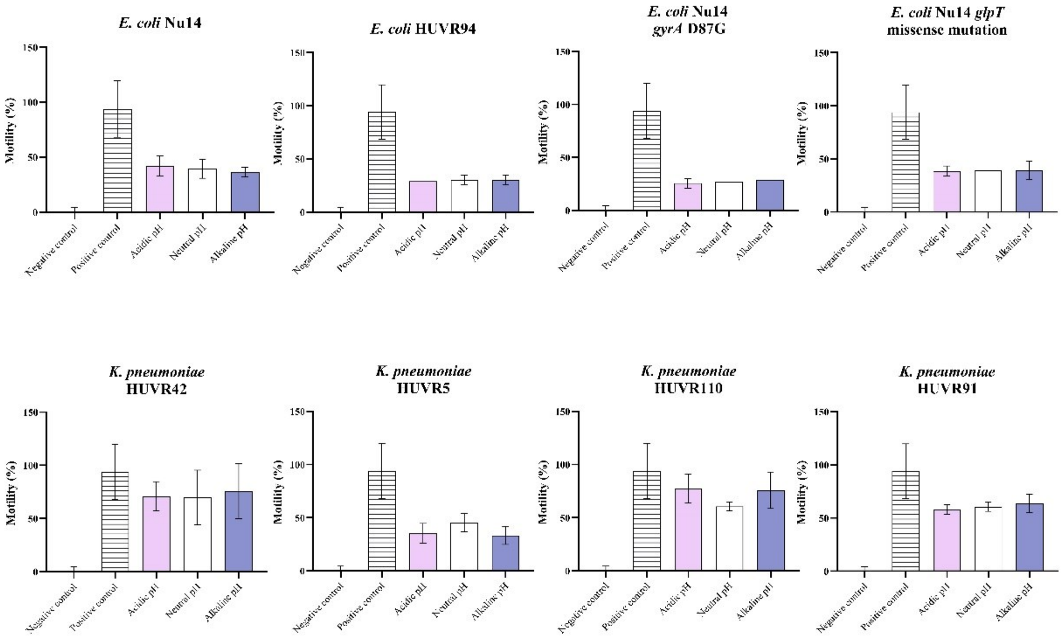

B)

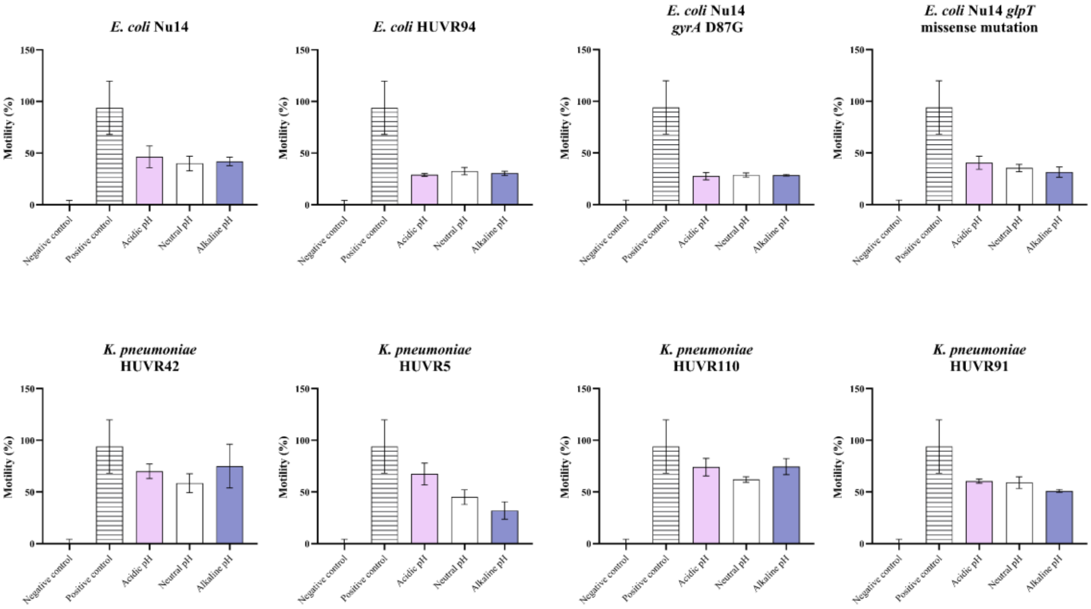

Figure S5. Motility of *Escherichia coli* and *Klebsiella pneumoniae* strains in Luria-Bertani (**A**) and filter-sterilized normal human urine (**B**) at different pH conditions. Stripped bar: control positive; Pink bar: Acidic pH; White bar: Neutral pH; Purple bar: Alkaline pH.

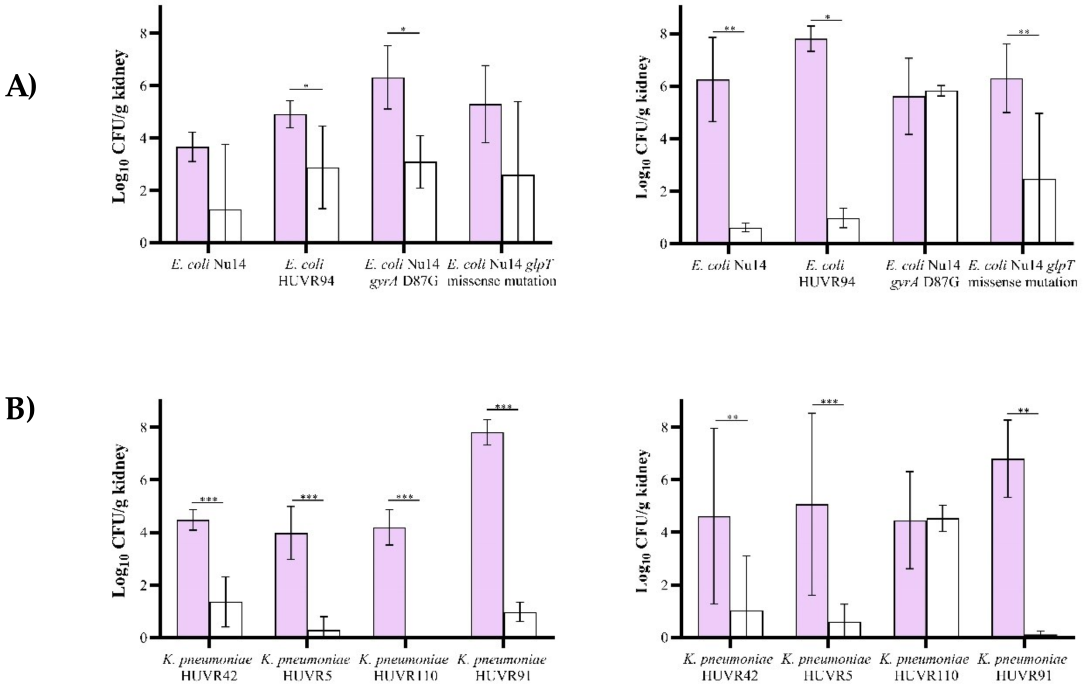

Supplementary Figure S6. Bacterial concentration in kidneys in murine urinary tract infection caused *Escherichia coli* (A) and *Klebsiella pneumoniae* (B) strains, at acidic and neutral urine pH. Kidneys bacterial concentration after 48 hours of infection, expressed as mean ± standard deviation, caused by *Escherichia coli* (**A**) or *Klebsiella pneumoniae* (**B**) in both immunocompetent (left) and immunosuppressed (right) murine model of UTI. Pink bar: Acidic urine pH; White bar: Neutral urine pH. *: P<0.05; **: P<0.01; ***: P<0.001.

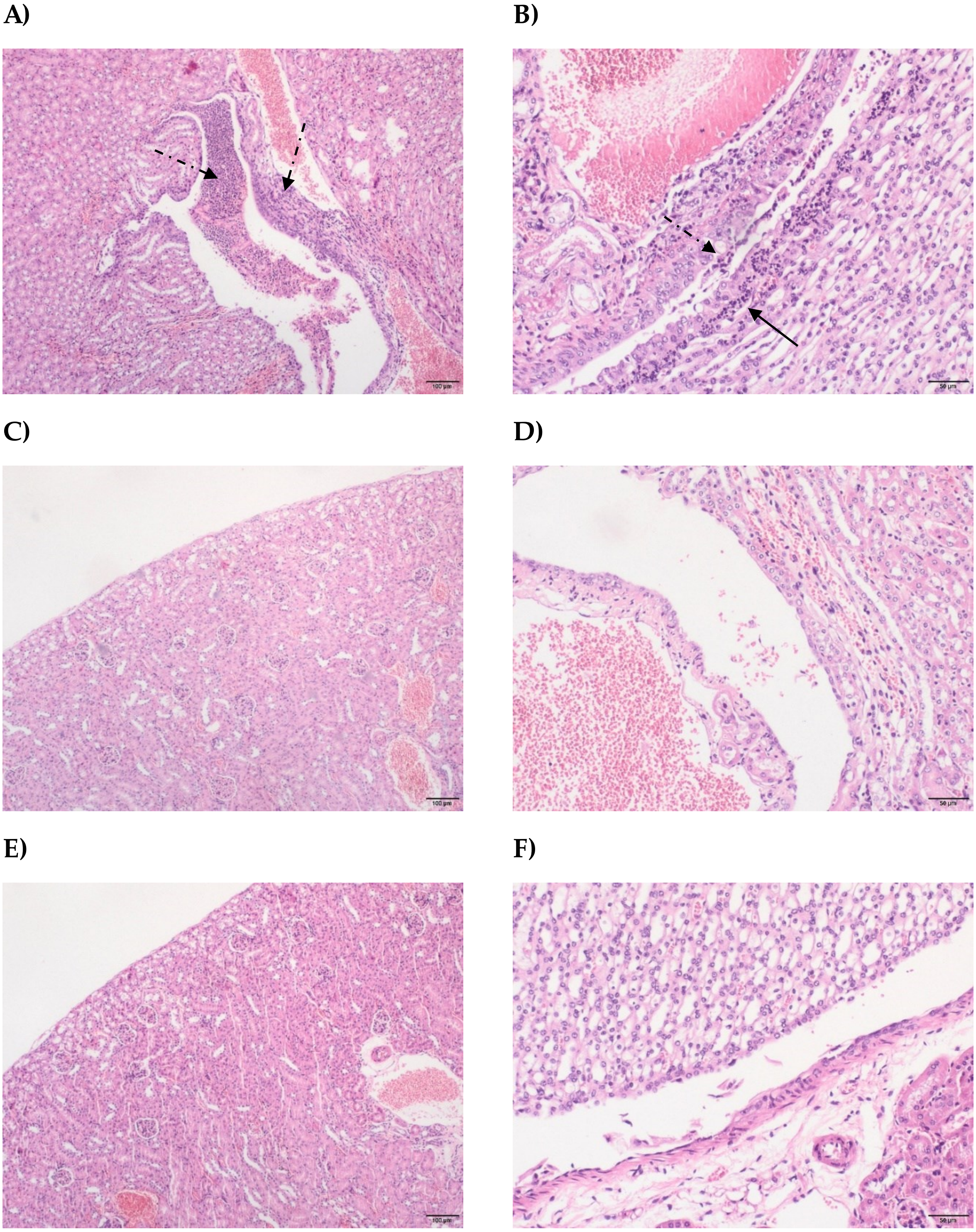

Supplementary Figure S7. Kidneys pathological findings in mice inoculated with 50 μL of 2 × 10^9 CFU/mL *E. coli* Nu14 at acidic urine pH (**A**,**B**), neutral urine pH (**C**,**D**), and alkaline urine pH (**E**,**F**). Hematoxylin-eosin staining x5 (**A**,**C**,**E**) and x10 (**B**,**D**,**F**). Arrow: Inflammatory infiltrate with

polymorphonuclear cells extending through the connective tissue beneath the transitional epithelium at the level of the renal pelvis. Dashed arrow: inflammatory cellularity in the lumen of the urinary tract (**A**,**B**).

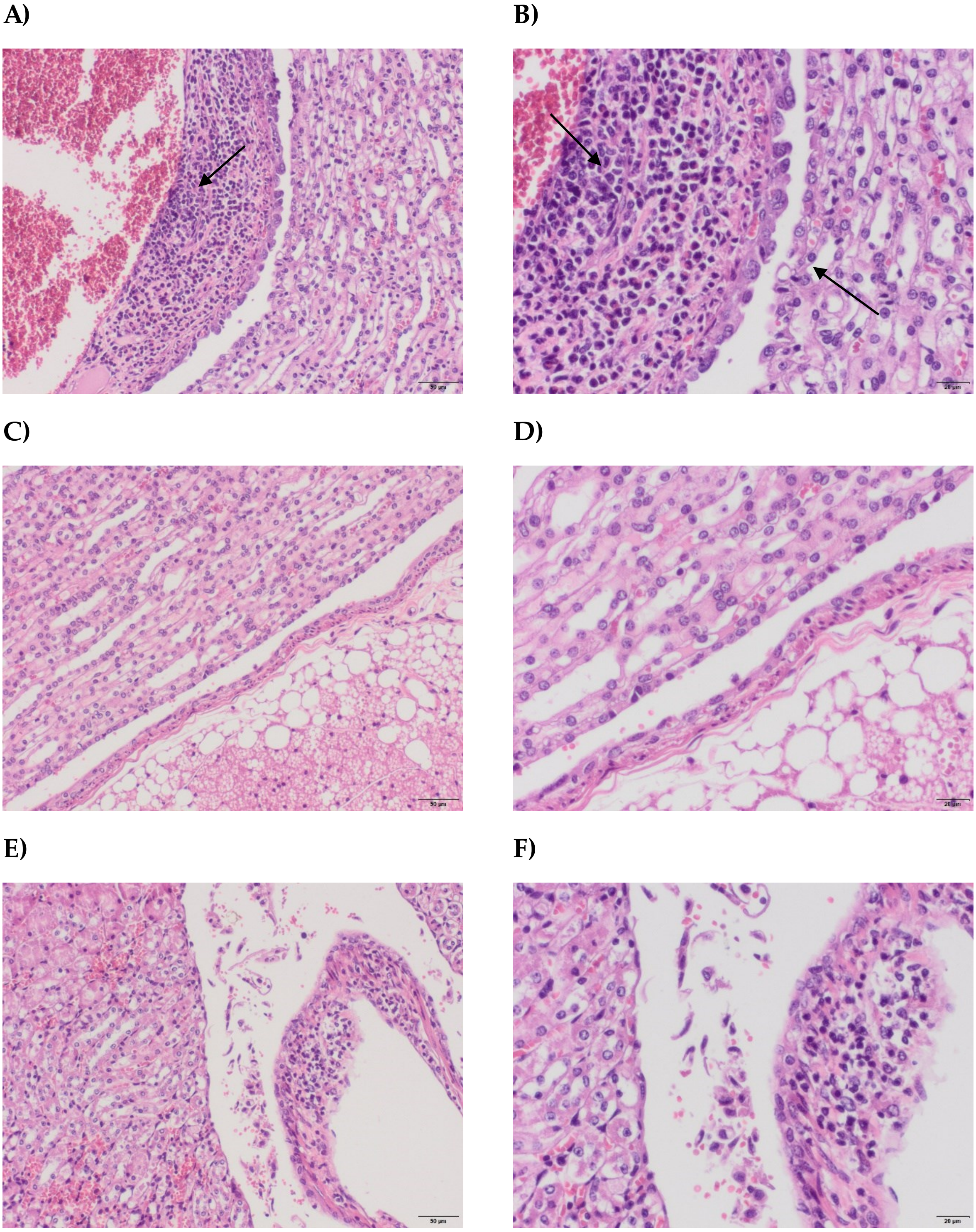

Supplementary Figure S8. Kidneys pathological findings in mice inoculated with 50 μL of 10^8 CFU/mL *K. pneumoniae* HUVR42 at acidic urine pH (**A**,**B**), neutral urine pH (**C**,**D**), and alkaline urine pH (**E**,**F**). Hematoxylin-eosin staining x10 (**A**,**C**,**E**) and x20 (**B**,**D**,**F**). Arrow: Inflammatory infiltrate with polymorphonuclear cells at the level of the renal pelvis, almost all the inflammatory cellularity is under the epithelium (**A**,**B**).
